# *ELOA3*: A primate-specific RNA polymerase II elongation factor encoded by a tandem repeat gene cluster

**DOI:** 10.1126/sciadv.adj1261

**Published:** 2023-11-22

**Authors:** Marc A. J. Morgan, Saeid Mohammad Parast, Marta Iwanaszko, Yuki Aoi, DongAhn Yoo, Zachary J. Dumar, Benjamin C. Howard, Kathryn A. Helmin, Qianli Liu, William R. Thakur, Jacob M. Zeidner, Benjamin D. Singer, Evan E. Eichler, Ali Shilatifard

**Affiliations:** ^1^ Department of Biochemistry and Molecular Genetics, Simpson Querrey Institute for Epigenetics, Northwestern University Feinberg School of Medicine, Chicago, IL 60611, USA.; ^2^ Department of Genome Sciences, University of Washington School of Medicine; Seattle, WA 98195, USA.; ^3^ Division of Pulmonary and Critical Care Medicine, Department of Medicine, Simpson Querrey Lung Institute for Translational Science, Northwestern University Feinberg School of Medicine, Chicago, IL 60611, USA.; ^4^ Howard Hughes Medical Institute, University of Washington, Seattle, WA 98195, USA.

## Abstract

The biological role of the repetitive DNA sequences in the human genome remains an outstanding question. Recent long-read human genome assemblies have allowed us to identify a function for one of these repetitive regions. We have uncovered a tandem array of conserved primate-specific retrogenes encoding the protein Elongin A3 (ELOA3), a homolog of the RNA polymerase II (RNAPII) elongation factor Elongin A (ELOA). Our genomic analysis shows that the *ELOA3* gene cluster is conserved among primates and the number of *ELOA3* gene repeats is variable in the human population and across primate species. Moreover, the gene cluster has undergone concerted evolution and homogenization within primates. Our biochemical studies show that ELOA3 functions as a promoter-associated RNAPII pause-release elongation factor with distinct biochemical and functional features from its ancestral homolog, ELOA. We propose that the *ELOA3* gene cluster has evolved to fulfil a transcriptional regulatory function unique to the primate lineage that can be targeted to regulate cellular hyperproliferation.

## Teaser

A previously undescribed primate-specific gene cluster encodes a positive regulator of RNA polymerase II transcription elongation.

## INTRODUCTION

A large fraction of the human genome is composed of repetitive DNA, often derived from transposable elements (TEs) (*1*, *2*). Studies to date suggest that these sequences have complex biological functions and play an important role in genetic diversity and evolution (*3*–*5*). However, because of their repetitive nature, studying these regions presents a considerable challenge to short-read DNA sequencing technologies. For decades, the genetic structure of many regions of the human genome remained undetermined, until long-read DNA sequencing approaches empowered the first complete telomere-to-telomere (T2T) human genome assemblies (*6*–*9*). These assemblies not only determined the sequence of extended repetitive chromosomal structures such as centromeres and telomeres but have also uncovered clustered tandem repeats of ORFs that likely encode uncharacterized human proteins, thus opening new frontiers for the study of human genetics and molecular biology (*6*–*8*).

While performing a CRISPR-Cas9 screen for resistance to the BET bromodomain inhibitor (BETi), JQ1 (*10*), we unexpectedly identified a primate-specific tandem repeat containing a cluster of genes encoding *Elongin A3* (*ELOA3*), a recently evolved homolog of the RNA polymerase II (RNAPII) elongation factor, *Elongin A* (*ELOA*). The ancestral ELOA protein was initially characterized as a factor that can stimulate processive transcriptional elongation by RNAPII in cell-free in vitro transcription systems in a complex with two additional proteins, Elongin B (ELOB) and Elongin C (ELOC) (*11*–*13*). In addition to its potential role in RNAPII elongation, ELOA forms a ubiquitin ligase complex with CULLIN-5 (CUL5) via the ELOB and ELOC adaptor proteins and is implicated in RNAPII ubiquitination in response to stress signals (*14*, *15*). Although cell-free in vitro studies support a role for ELOA in regulating the processive elongation activity of RNAPII, in vivo studies have yielded conflicting results (*16*–*18*). *Eloa* knockout mice survive until midgestation (*17*), indicating that the protein is not generally required for transcriptional elongation control. Moreover, recent studies in both mouse *Eloa* knockout and human *ELOA* knockdown cell lines observed minimal alterations in transcription (*16*, *18*). Thus, it remains to be established whether ELOA functions primarily as a regulator of RNAPII elongation or as a ubiquitin ligase. An in vitro study of the properties of recombinant ELOA3 protein suggests that it may have similar properties to ELOA with respect to stimulating RNAPII elongation in cell-free systems, although its activity is not stimulated by ELOB and ELOC (*19*).

In this study, we report several remarkable features of the *ELOA3* gene cluster locus and the protein that it encodes. We demonstrate that *ELOA3* is encoded by a primate-specific tandem repeat located on human chromosome 18q21. The *ELOA3* gene cluster is conserved among primates, and the number of *ELOA3* gene repeats is variable across the human population and between primate species. Our analysis suggests that the *ELOA3* gene cluster has undergone concerted evolution and homogenization within the primates. In the context of BETi, ELOA3 protein triggers the specific transcriptional activation of a subset of genes, including many immediate early genes. Biochemical purifications and molecular comparison of the ELOA3 and ELOA protein complexes and chromatin localization revealed that these proteins participate in separate RNAPII protein complexes with distinct cellular functions. Protein domain–mapping experiments revealed that the ELOA3 C-terminal disordered domain is essential for its ability to interact with RNAPII and induce transcriptional activation. These findings advance our understanding of the molecular genetics of the *ELOA3* gene cluster and, in contrast to cell-free in vitro systems, demonstrate that ELOA3 and ELOA proteins form distinct RNAPII complexes with divergent regulatory functions in human cells. These findings have broad-reaching implications for the fields of transcriptional regulation, primate evolution, and the study of repetitive DNA sequences.

## RESULTS

### A tandem array of primate-specific retrogenes encode Elongin A3

**Fig. 1.**
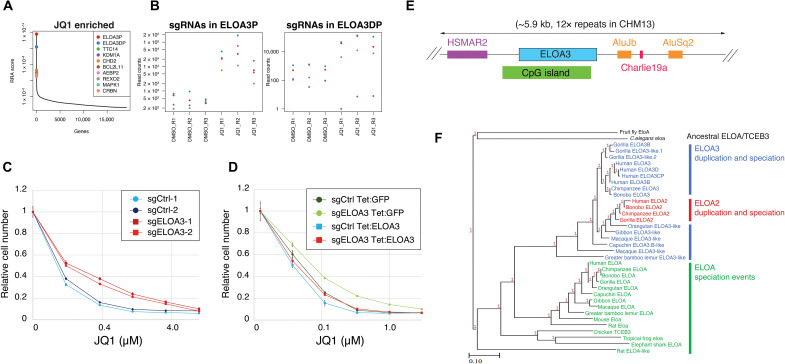
A tandem array of primate-specific retrogenes encode Elongin A3. (**A**) Summary of MAGeCK robust ranking aggregation (RRA) scores for sgRNA enriched in JQ1-treated cells relative to DMSO control. (**B**) Sequencing read counts for four independent sgRNAs targeting (left) *ELOA3P* and (right) *ELOA3DP*, displaying three replicates of DMSO- and JQ1-treated samples. (**C**) JQ1 dose-response experiment in control and *ELOA3* sgRNA–treated SYO-1 cells. Data represent means ± SD, *n* = 3. (**D**) JQ1 dose-response experiment in control and *ELOA3* sgRNA–treated cells expressing either a tetracycline-inducible GFP (Tet:GFP) control or ELOA3 (Tet:ELOA3) construct. Data represent means ± SD, *n* = 3. (**E**) Schematic of the structure of the *ELOA3* gene cluster. In the CHM13 genome assembly, 12 repeats of the ELOA3 coding sequence are flanked by *Homo sapiens* mariner 2 (HSMAR2), AluSq2, AluJb, and Charlie19a repetitive elements. (**F**) Phylogenetic analysis of the *ELOA*, *ELOA2*, and *ELOA3* genes. Gene sequences for representative species were aligned using webPRANK, with default gap parameters and relaxed substitution scoring.

To investigate the function of the transcription elongation regulator BRD4 (*20*, *21*) in controlling gene expression and cellular proliferation, we performed a genome-wide CRISPR-Cas9 screen for resistance to the BETi JQ1 (*10*) in the SYO-1 human cell line. We chose to use the SYO-1 cell line because of its low mutational burden (*22*), on the rationale that performing our screen in a genetic background with a large number of pre-existing mutations could mask biologically relevant targets. When we compared JQ1-treated cells to dimethyl sulfoxide (DMSO) control, we observed an enrichment of single-guide RNAs (sgRNAs) targeting *ELOA3P* and *ELOA3DP* (Fig. 1, A and B). These two loci are annotated as pseudogenes but contain ORFs with the potential to encode identical proteins. To validate our screen, we infected SYO-1 cells with two independent sgRNAs that simultaneously target both genes (hereafter collectively referred to as *ELOA3*) and performed BETi dose-response experiments (Fig. 1C and fig. S1, A to C). SYO-1 cells expressing *ELOA3*-targeting sgRNAs are more resistant to JQ1 as well as two additional BETi (OTX-015 and ABBV-744) compared to cells infected with control sgRNAs; however, no difference in survival is observed in response to treatment with doxorubicin (Fig. 1C and fig. S1, A to C). This suggests that ELOA3 depletion confers specific resistance to BETi treatment rather than causing a general increase in cellular fitness. To examine whether this effect is mediated by loss of the ELOA3 protein, we re-expressed ELOA3 from an sgRNA-resistant tetracycline-regulated construct (Tet:ELOA3) (Fig. 1D and fig. S1D). Expression of ELOA3 but not a green fluorescent protein (GFP) control (Tet:GFP) restores JQ1 sensitivity to *ELOA3*-deficient cells (Fig. 1D and fig. S1D), thus confirming ELOA3 protein as a mediator of JQ1 sensitivity in this cell line.

In the hg38 human genome assembly, two small clusters of *ELOA3* genes are separated by a ~50,000–bp gap of undetermined DNA sequence. An examination of the recently published T2T CHM13 genome assembly revealed 12 nearly identical *ELOA3* tandem repeats spanning this sequence gap (*7*). Each of these repeat units contains a *Homo sapiens mariner 2* (*HSMAR2*) TE upstream of the CpG-rich *ELOA3* coding exon followed by three additional repetitive elements (*AluJb*, *Charlie19a*, and *AluSq2*) (Fig. 1E). Whereas *ELOA* is encoded by a multiexon spliced transcript, *ELOA3* is an intronless gene suggesting that *ELOA3* is retrogene. Phylogenetic analysis revealed that *ELOA3* is found only in primates, whereas *ELOA* homologs were detected in all the metazoan species that we examined (Fig. 1F).

**Fig. 2.**
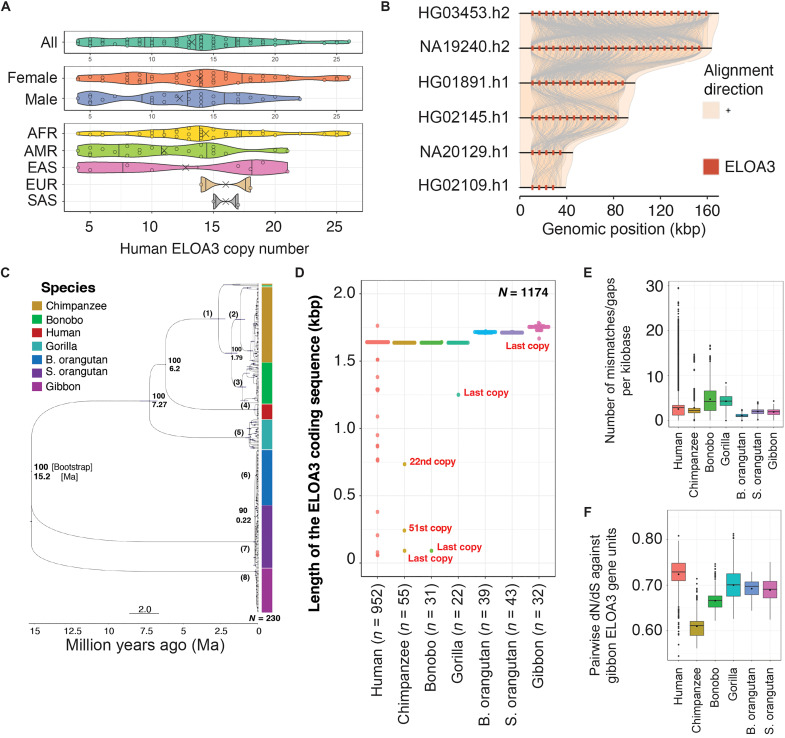
Structural comparison of the human and primate *ELOA3* gene cluster. (**A**) Violin plot of the distributions of *ELOA3* gene copy numbers across subsets of the human population based on 71 human haplotype genome assemblies. AFR, African; AMR, Admixed American; EAS, East Asian; EUR, European; SAS, South Asian. (**B**) Alignment of human *ELOA3* gene arrays from six representative genome assemblies with the copy number ranging from 4 to 26. Red bars indicate the position of the *ELOA3* protein coding sequence. (**C**) A maximum likelihood tree of human and primate *ELOA3* coding sequences. Bootstrap value and time-calibrated divergence are indicated. The gene family expansions were estimated with 95% confidence: (1) chimpanzee/bonobo 2.66 (2.19 to 3.23), (2) chimpanzee 1.11 (0.87 to 1.53), (3) bonobo 1.06 (0.81 to 1.37), (4) human 0.21 (0.085 to 0.33), (5) gorilla 0.61 (0.45 to 0.84), (6) B. orangutan 0.13 (0.088 to 0.18), (7) S. orangutan 0.22 (0.15 to 0.33), and (8) gibbon 0.15 (0.084 to 0.26) Ma. (**D**) A length distribution comparison of the *ELOA3* coding sequences in nucleotide base pairs for human versus nonhuman ape species. The number of gene copies analyzed for each species are shown in parentheses.(**E**) Genetic variation (number of mismatch/gap opening) per 1 kb of sequence in each species. The significant Wilcox *P* value (<0.05) against human is indicated by an asterisk. (**F**) Pairwise dN/dS distribution of *ELOA3* gene family using gibbon as an outgroup signifies negative selection in all species (dN/dS < 1.0).

To investigate the evolutionary history and copy number of *ELOA3* gene repeats in humans and nonhuman apes, we analyzed long-read sequence assemblies of representative genomes from seven primate species resolving 230 putative full-length *ELOA3* copies across 20 million years of primate evolution as well as 71 human haplotype-resolved genomes from the Human Pangenome Reference Consortium (HPRC) (*9*, *23*) (Fig. 2A and fig. S2, A and B). For each primate species, both haplotypes were remarkably consistent, apart from the Sumatran orangutan, where the two haplotypes are predicted to differ by 24 copies (67 versus 43 copies) (fig. S2A). On the basis of the CHM13-T2T and 71 HPRC human haplotype assemblies, humans show a reduced number of *ELOA3* repeats relative to nonhuman primates (fig. S2A). Human haplotype copy number ranges from 4 to 26 copies with most carrying 13 to 16 copies of *ELOA3*, whereas the primate haplotypes range from 22 copies (gorilla) to 67 (Sumatran orangutan) (Fig. 2, A and B, and fig. S2, A and B). These findings suggest a marked reduction in the number of human *ELOA3* gene family members since the divergence from apes 7 to 10 million years ago (Ma).

We constructed a maximum likelihood phylogenetic tree of 230 *ELOA3* primate and human genes (Fig. 2C). The resulting topology revealed a near-complete separation of *ELOA3* copies consistent with the species tree (bootstrap 90 to 100), with the exception of one copy (Fig. 2C) that was predicted to be ancestral between chimpanzee and bonobo. These results suggest either independent duplication or recurrent gene conversion occurring within each primate lineage effectively homogenizing the sequence content in each primate lineage. We estimated the timing of each *ELOA3* copy number expansions using two species divergence calibration time points, namely human-chimpanzee (6.2 Ma) and human-orangutan (15.2 Ma). The analyses predict very recent expansion or gene conversion in all primate lineages. Most ape species show very recent homogenization of the locus with humans occurring in the last 100 to 200 thousand years.

Preservation of the *ELOA3* open reading frame (ORF) among primates would suggest a functional requirement for the protein, whereas the introduction of frameshift or nonsense codons would support pseudogenization. To evaluate these possibilities, we extracted the predicted ORF for each primate and found that 97% of the copies (228 of 234) maintain an ORF consistent with the human gene model (Fig. 2D and fig. S2A). Similarly, among the 952 human *ELOA3* copies examined, we find that 93% (*n* = 883) maintain an intact ORF strongly suggesting that the gene family has not been pseudogenized (Fig. 2D). Most of the truncated copies are positioned at the edge of the expanded gene cluster (Fig. 2D), and these edge copies, in general, show greater variability in structure consistent with variable number tandem repeat degradation. Using these data, we assessed different models of selection and found that all lineages showed evidence of modest purifying selection (dN/dS < 1.0) noting the chimpanzee showed the strongest signal despite having some of the highest copy number estimates (Fig. 2, E and F). We conclude that each primate lineage has evolved and homogenized distinct repertoires of *ELOA3* genes that have acquired a suite of amino acid replacements that are diagnostic to each of the ape lineages (fig. S2, C and D). In the human lineage, there are at least six diagnostic amino acid replacements of which four have been fixed in all ~900 copies examined (fig. S2, C and D).

In SYO-1 cells, the *ELOA3* gene cluster is transcriptionally silent; however, *ELOA3* transcripts become expressed after JQ1 treatment (fig. S3A). We tested several other cell lines for activation of *ELOA3* expression upon JQ1 treatment and found this effect to be limited to SYO-1 cells (fig. S3B). Thus, *ELOA3* is not a general mediator of sensitivity to BETi, but rather, this feature is a characteristic of the SYO-1 cell line. Given the high density of CpG dinucleotides at the *ELOA3* cluster, we hypothesized that it might be silenced by DNA cytosine methylation. Reduced representation bisulfite sequencing revealed that the *ELOA3* cluster has high levels of DNA methylation in cell lines that do not activate ELAO3 in response to JQ1 but is hypomethylated in SYO-1 cells (fig. S3B). In support of this, treatment with the DNA methyltransferase inhibitor 5-aza-2′-deoxycytidine (decitabine) results in *ELOA3* expression in a subset of the cell lines we tested (fig. S3, B and D).

### Identification of ELOA3 transcriptional targets in human cells

**Fig. 3.**
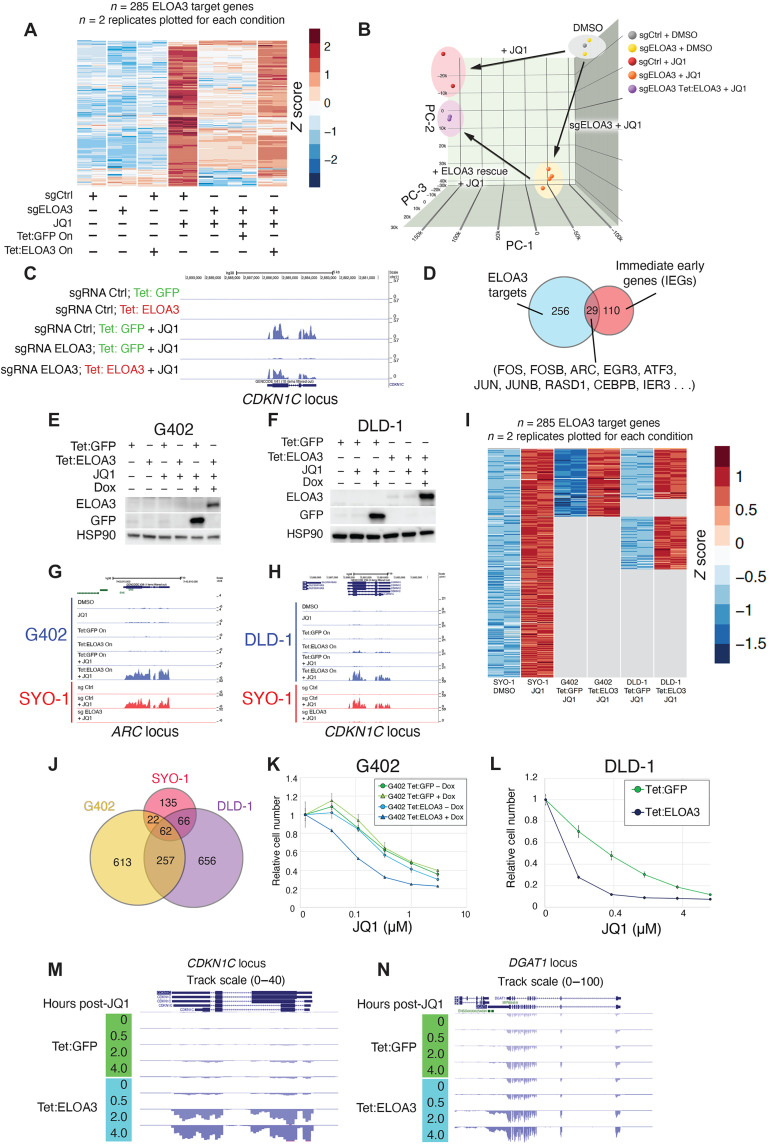
Identification of ELOA3 transcriptional targets in human cells. (**A**) RNA-seq heatmap plotting *z* scores for log_2_-transformed counts for ELOA3-dependent genes in control and *ELOA3* sgRNA–treated cells expressing Tet:GFP or Tet:ELOA3 constructs and treated with DMSO or JQ1 (1 μM) for 48 hours. (**B**) Three-dimensional multidimensional scaling (3D-MDS) plot of RNA-seq data from control and *ELOA3* sgRNA–treated cells and expressing Tet:GFP or Tet:ELOA3 constructs and treated with DMSO or JQ1 (1 μM) for 48 hours. Axis units indicate component scores. (**C**) RNA-sequencing track example of an ELOA3 target gene (*CDKN1C*) whose expression is rescued by re-expression of ELOA3. (**D**) Venn diagram of ELOA3-dependent genes and immediate early genes (IEGs). (**E** and **F**) Immunoblots of whole cell extracts from (E) G402 cells and (F) DLD-1 cells expressing Tet:GFP control or Tet:ELOA3 and treated with DMSO or JQ1. (**G** and **H**) RNA-seq track examples of ELOA3 target genes in (G) G402 and (H) DLD-1 cells expressing Tet:GFP or Tet:ELOA3 and treated with DMSO or JQ1 (1 μM) for 48 hours. (**I**) RNA-seq heatmap plotting *z* scores for log_2_-transformed counts for genes that are activated by ELOA3 in SYO1 cells, displaying data for G402 and DLD-1 cells expressing Tet:GFP or Tet:ELOA3 and treated with JQ1 (1 μM) for 48 hours. (**J**) Venn diagram of genes that are dependent on ELOA3 for activation in SYO-1, G402, and DLD-1 cells. (**K** and **L**) JQ1 dose-response experiments performed in (K) G402 and (L) DLD-1 cells expressing Tet:GFP or Tet:ELOA3. Data represent means ± SD, *n* = 3. (**M** and **N**) RNA-seq track examples displaying (M) the *CDKN1C* and (N) DGAT1 locus in *DLD*-1 cells expressing Tet:GFP or Tet:ELOA3 and subjected to a JQ1 treatment time course for 0, 0.5, 2, or 4 hours.

Given the homology between ELOA3 and ELOA, we examined whether ELOA3 protein might play a role in transcriptional regulation. To this end, we performed RNA sequencing (RNA-seq) experiments in control and *ELOA3* sgRNA cells treated with DMSO or JQ1 and expressing either Tet:GFP or the Tet:ELOA3 rescue construct (Fig. 3, A to D). Treatment of SYO-1 cells with JQ1 results in up-regulation of 2390 transcripts and down-regulation of 2906 transcripts. Of the JQ1 up-regulated genes, 153 display down-regulation (log_2_ fold change < −1.5) in *ELOA3* sgRNA cells, whereas for JQ1 down-regulated genes, only 9 show up-regulation (log_2_ fold change >1.5) in *ELOA3* sgRNA–treated cells. These data suggest that ELOA3 functions primarily as a transcriptional activator for a subset of JQ1 activated genes, and therefore, we chose to focus on genes activated by JQ1 in control sgRNA cells that fail to up-regulate in *ELOA3* sgRNA–treated cells (Fig. 3A). In total, we identified 285 genes that fail to activate upon JQ1 treatment in *ELOA3* sgRNA cells and can be rescued by re-expression of Tet:ELOA3 (Fig. 3, A and B). Intriguingly, Tet:ELOA3 expression in control sgRNA cells absent JQ1 treatment is insufficient to trigger expression of this gene set; thus, these genes require both the presence of ELOA3 protein and JQ1 treatment for activation (Fig. 3, A and C). Notably, the gene set activated by ELOA3 contains several immediate early genes with roles in stress responses and developmental processes, such as *ARC*, *FOS*, *FOSB*, *JUN*, *JUN*, and *EGR3* (Fig. 3D) (*24*).

Although activation of the *ELOA3* gene cluster by JQ1 treatment is unique to SYO-1 cells (fig. S3), we speculated that exogenously expressed ELOA3 protein might show a similar ability to alter gene expression in combination with JQ1 treatment in other cell types. To test this possibility, we expressed Tet:ELOA3 or Tet:GFP in G402 (malignant rhabdoid tumor) and DLD-1 (colorectal adenocarcinoma) cells, treated them with DMSO or JQ1, and performed RNA-seq experiments (Fig. 3, E to J). These experiments revealed that, akin to SYO-1 cells, ELOA3 protein in DLD-1 and G402 cells triggers the expression of a subset of genes when combined with JQ1 treatment; neither ELOA3 expression nor JQ1 treatment individually is sufficient to trigger the expression of these targets (Fig. 3, G and H). We next examined whether there were any common target genes shared between SYO-1 cells and these two additional cell lines. More than half of ELOA3 targets in SYO-1 cells are also targets in either G402 or DLD-1 cells (Fig. 3, I and J). To determine whether these altered patterns of gene expression correlate with a change in cellular phenotype, we performed JQ1 dose-response experiments in G402 and DLD-1 cells expressing Tet:GFP or Tet:ELOA3 and observed that the presence of ELOA3 protein renders these cells more sensitive to JQ1 treatment (Fig. 3, K and L). Thus, ELOA3 can trigger the activation of a common gene set across several cell types in response to BETi, and this is associated with an increase in cellular sensitivity to BETi.

Because ELOA3 regulates the mRNA expression of several transcription factors that could mediate secondary effects on gene expression, we performed RNA-seq at early time points after JQ1 treatment to determine early targets of ELOA3. DLD-1 cells expressing GFP control or ELOA3 were subjected to JQ1 treatment for 30 min, 2 hours, or 4 hours. We observed activation of similar ELOA3 targets to longer treatments, including *CDKN1C* and *DGAT1*, which activate between 30 min and 2 hours after JQ1 treatment (Fig. 3, M and N), suggesting that these genes are immediate direct targets of ELOA3.

### ELOA3 and ELOA form distinct RNAPII complexes

**Fig. 4.**
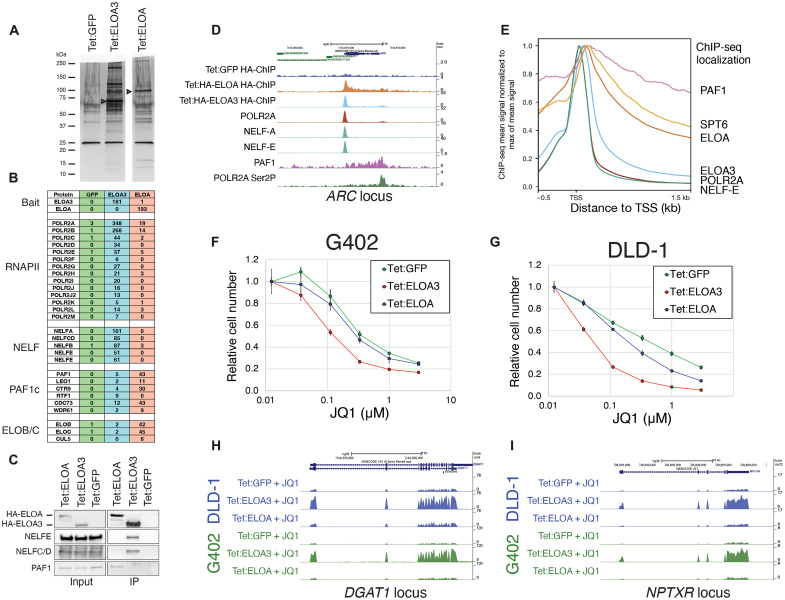
ELOA3 and ELOA form distinct RNAPII complexes. (**A**) Silver-stained SDS-PAGE gel of anti-Flag immunoprecipitations from Tet:GFP control, Tet:ELOA3-, and Tet:ELOA-expressing SYO-1 cells. (**B**) Table of mass spectrometry spectral counts from the samples shown in (A). (**C**) Western blots of inputs and immunoprecipitations from SYO-1 cells expressing Tet:GFP control, Tet:ELOA3, or Tet:ELOA probed with the antibodies indicated. (**D**) ChIP-seq track example of an ELOA3 target gene *ARC* in SYO-1 cells displaying data for the antibodies indicated. Cells were treated with 1 μM JQ1 for 24 hours. (**E**) Genome-wide occupancy plot centered on transcription start sites (TSS) for the ChIP-seq samples indicated in SYO-1 cells treated with 1 μM JQ1 for 24 hours. For each ChIP-seq sample, ChIP-seq mean signals (*N* = 10,033 expressed genes) were normalized such that the maximum value of mean signal at the indicated regions is equal to 1. (**F** and **G**) JQ1 dose-response experiments performed in (F) G402 and (G) DLD-1 cells expressing Tet:GFP, Tet:ELOA3, or Tet:ELOA. Data represent means ± SD, *n* = 3. (**H** and **I**) RNA-seq track examples of ELOA3 target genes in (blue) DLD-1 and (green) G402 cells at the (H) *DGAT1* and (I) *NPTXR* gene loci in cells expressing the constructs indicated and treated with JQ1 (1 μM) for 48 hours.

To investigate the biochemical properties of ELOA3 relative to ELOA, we expressed epitope-tagged ELOA3, ELOA, or GFP control in SYO-1 cells and performed immunoprecipitation from nuclear extracts (Fig. 4A). Mass spectrometric analyses and Western blotting of immunoprecipitated proteins revealed that ELOA3 and ELOA form distinct RNAPII complexes (Fig. 4, B and C). ELOA3 interacts with RNAPII and the negative elongation factor (NELF) complex but shows minimal interaction with the polymerase associated factor 1 complex (PAF1c) (Fig. 4, B and C). Conversely, ELOA enriches multiple subunits of PAF1c but shows minimal enrichment of NELF in comparison to ELOA3 (Fig. 4, B and C). In vitro studies have suggested that both ELOA and ELOA3 interact with the cofactors ELOB and ELOC (*13*, *19*, *25*). However, our biochemical purifications revealed that only ELOA interacts with ELOB and ELOC (spectral counts of 42 and 45, respectively), whereas ELOB and ELOC are present at near-background levels in ELOA3 purifications (spectral counts of 2 for both proteins in ELOA3 IPs versus 1 in the GFP control) (Fig. 4B). On the basis of our proteomics data indicating that ELOA and ELOA3 associate with different RNAPII complexes, we hypothesized that these proteins might display different localization patterns on chromatin. To test this, we performed chromatin immunoprecipitation next-generation sequencing (ChIP-seq) for epitope-tagged ELOA3 and ELOA (Fig. 4, D and E, and fig. S4, A to D). Consistent with our biochemical data, ELOA3 displays preferential colocalization with the NELF complex at promoter-proximal gene regions, whereas ELOA displays localization to gene bodies associated with elongating RNAPII and PAF1c (Fig. 4, D and E). ChIP-seq performed with an antibody against endogenous ELOA3 in SYO1 cells revealed a distribution pattern very similar to our epitope-tagged ELOA3 ChIP-seq, exhibiting colocalization at gene promoters with RNAPII (fig. S4, A to D).

We next tested whether ELOA3 and ELOA have different cellular functions with respect to BETi sensitivity. Consistent with our previous data, ELOA3 increases the sensitivity of both the G402 and DLD-1 cell lines to JQ1 treatment (Fig. 4, F and G). However, experiments performed in parallel with a Tet:ELOA expression construct revealed that ELOA causes only a modest increase in JQ1 sensitivity (Fig. 4, F and G). Moreover, transcriptomic analysis revealed that ELOA does not trigger the activation of ELOA3 targets in either of these cell lines (Fig. 4, H and I). Collectively, these experiments demonstrate that ELOA3 and ELOA form distinct biochemical complexes with divergent cellular and transcriptional targets/activities. This stands in contrast to findings from in vitro, cell-free systems, which had previously suggested that ELOA and ELOA3 form identical complexes with similar transcriptional activities (*19*).

### The ELOA3 C-terminal disordered domain is required for its function

**Fig. 5.**
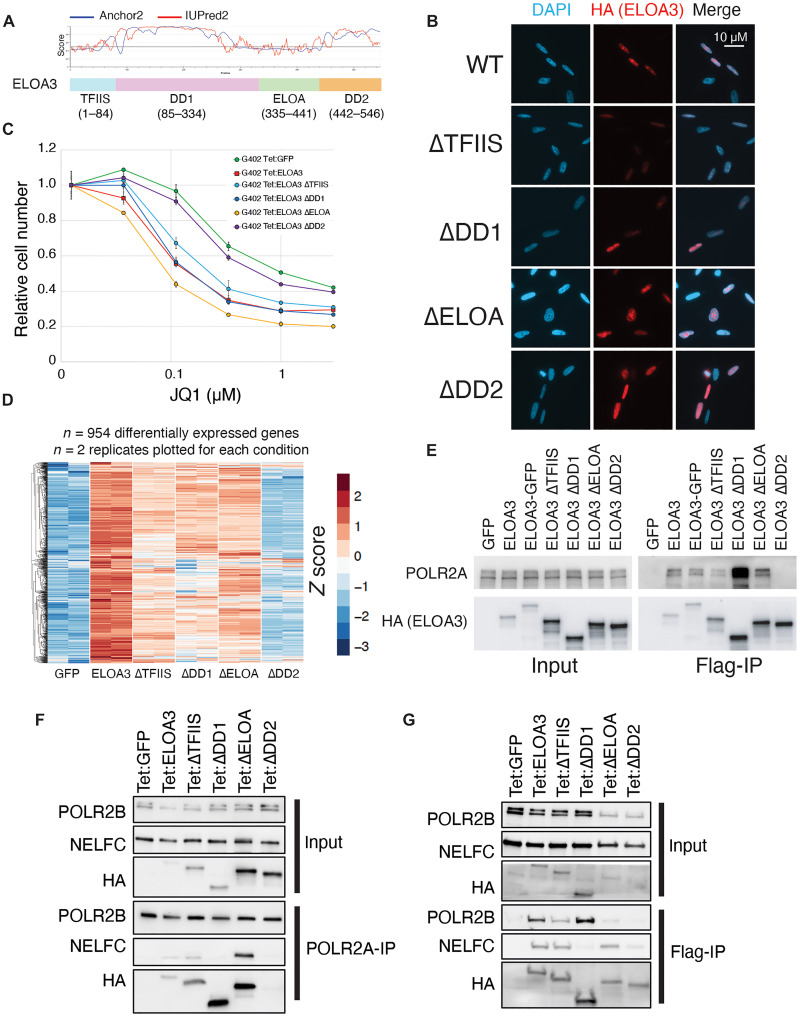
The ELOA3 C-terminal disordered domain is required for its function. (**A**) Diagram of the ELOA3 protein domains plotting disorder prediction using IUPred2A and Anchor2. TFIIS, Transcriptional factor II S homology domain; DD1, disordered domain 1; ELOA, Elongin A homology domain; DD2, disordered domain 2. (**B**) Anti-HA immunofluorescence of wild-type and ELOA3 truncation constructs lacking the domains indicated. (**C**) JQ1 dose-response experiments in G402 cells expressing wild-type or truncated ELOA3 expression constructs. Data represent means ± SD, *n* = 3. (**D**) RNA-seq heatmap plotting *z* scores for log_2_-transformed counts displaying genes that are activated in G402 cells by the combination of wild-type Tet:ELOA3 expression and JQ1 treatment. (**E**) Immunoblots of input samples anti-Flag immunoprecipitations of wild-type or truncated ELOA3 expression constructs probed with the antibodies indicated. (**F**) Immunoblots of input samples anti-POLR2A immunoprecipitations of wild-type or truncated ELOA3 expression constructs probed with the antibodies indicated. (**G**) Immunoblots of input samples anti-Flag immunoprecipitations of wild-type or truncated ELOA3 expression constructs probed with the antibodies indicated.

The ELOA3 protein can be divided into four regions: an N-terminal TFIIS domain (TFIIS), a central disordered domain (DD1), an Elongin A homology domain (ELOA), and a C-terminal disordered domain (DD2) (Fig. 5A). We generated ELOA3 deletion constructs lacking each of these regions for cellular and biochemical assays. Initially, we performed immunofluorescence experiments and found that each of these deletion mutants localizes to the nucleus (Fig. 5B). Deletion of the C-terminal disordered domain (∆DD2) fully abrogates the ability of ELOA3 to induce sensitivity to JQ1 in G402 cells (Fig. 5C). Consistent with this finding, RNA-seq experiments with these deletion mutants revealed that genes induced by ELOA3 in combination with JQ1 fail to activate in G402 cells expressing the ELOA3-∆DD2 mutant, whereas only modest reductions in gene activation are observed for the other mutants (Fig. 5D). Given the inability of ELOA3-∆DD2 to activate target gene expression, we examined the ability of our mutants to interact with RNAPII in coimmunoprecipitation assays (Fig. 5E). This experiment revealed that the ELOA3-∆DD2 mutant completely disrupts RNAPII binding, whereas all other deletion mutants retain binding to RNAPII (Fig. 5E). Together, these experiments demonstrate that the ELOA3 C-terminal DD2 region is indispensable for its interaction with RNAPII, as well as its ability to activate gene expression and induce JQ1 sensitivity. However, other domains may play subtle roles to fine-tune ELOA3 activity, as their deletion mutants display modestly reduced expression of ELOA3 targets (Fig. 5D). To explore how additional domains of ELOA3 might mediate binding partner interactions and influence RNAPII complexes, we performed reciprocal immunoprecipitations for ELOA3 and POLR2A in cells expressing ELOA3 truncation mutants (Fig. 5, F and G). Immunoprecipitation of POLR2A confirmed our finding that ELOA3 DD2 is critical for interaction with RNAPII (Fig. 5F). In addition, these experiments revealed that ELOA3 interaction with NELF requires the separate DD1 region of ELOA3 (Fig. 5, F and G).

## DISCUSSION

*ELOA3* exhibits unique attributes that distinguish it from its ancestral homolog *ELOA*. Whereas *ELOA* is ubiquitously expressed, the *ELOA3* gene cluster appears to be silent or very lowly expressed in most cell lines that we have examined. The SYO-1 cell line appears to be a unique outlier in which *ELOA3* expression is inducible by treatment with BETi, which likely explains why others have not identified *ELOA3* in screens for JQ1 resistance and sensitivity (*26*–*34*). While *ELOA3* is not a general mechanism for BETi sensitivity, we cannot rule out that this feature may exist in other cell lines or cancer types. Considering that *ELOA3* has been identified as a potential imprinted locus (*35*), the cluster may exhibit a restricted pattern of expression during embryonic development. Determining where, when, and how ELOA3 transcripts become expressed will be essential to unraveling its function in primates, to which its evolutionary conservation points. Considering that the *ELOA3* gene cluster is specific to primates, it is tempting to speculate that it may be involved in biological processes that show differences between primates and other mammals such as neurodevelopment, reproduction, and embryonic development.

In addition to differences in genetic regulation, the ELOA3 and ELOA proteins display evolutionary divergence in their biochemical activities and cellular functions. ELOA3 forms a biochemical interaction with RNAPII and the NELF complex and by ChIP-seq colocalizes to promoter-proximal regions along with both NELF and RNAPII. By contrast, ELOA does bind to NELF, but rather shows an interaction with PAF1c, and localizes to active gene bodies occupied by elongating RNAPII, in close agreement with recently published work in human cancer cells and mouse embryonic stem cells (*16*, *18*). Our laboratory recently reported the finding that the elongation factor SPT6 promotes the association of PAF1c with RNAPII and prevents NELF from interacting with elongating RNAPII (*36*); therefore, it will be important for future studies examining the relationship between these factors, ELOA3 and ELOA. In further support of the divergent function of these proteins, ELOA3 induces cellular sensitivity to BETi, whereas ELOA causes only a modest increase in sensitivity in one of the cell lines that we examined.

The C-terminal ELOA3 DD2 is essential for interaction with RNAPII and its transcriptional regulatory function. This finding stands in contrast to cell-free in vitro transcriptional systems that mapped ELOA RNAPII binding to the TFIIS domain and concluded that the ELOB/ELOC binding region as well as part of the C terminus was required for stimulation of RNAPII elongation (*25*, *37*). Moreover, in vitro studies conducted with recombinant ELOA3 produced in insect cells concluded that the protein has a similar function (*19*). However, our studies demonstrate that the crucial difference between these proteins is due to their formation of distinct RNAPII complexes in mammalian cells, a feature that escaped detected in reconstituted in vitro assays.

Precisely how ELOA3 influences RNAPII transcriptional activity at a unique set of genes is an important area for future studies. The association between ELOA3 and NELF containing RNAPII complexes may provide some insight into its function. Although long thought to be a negative regulator of RNAPII productive elongation, a recent study from our group revealed that NELF depletion does not result in widespread pause-release but rather disrupts assembly of RNA-processing factors with RNAPII (*38*). Thus, perhaps ELOA3 target genes may have unique requirements for RNA processing for their expression.

The genetic properties of *ELOA3* make it an intriguing locus for future studies, with wide-reaching relevance for the fields of transcriptional regulation, human/primate evolution, repetitive DNA elements, disease pathogenesis, and targeted therapeutics.

## MATERIALS AND METHODS

### Cell culture

Human embryonic kidney (HEK) 293T (CRL-3216), DLD-1 (CCL-221), and G402 (CRL-1440) cells were obtained from American Type Culture Collection. SYO-1 cells (*39*) were a gift from C. Kadoch. Cells were cultured in Dulbecco’s modified Eagle’s medium (Thermo Fisher Scientific, 11965-118) supplemented with 15% fetal bovine serum (MilliporeSigma, F2442), 1× GlutaMAX (Thermo Fisher Scientific, 35050-079), and penicillin-streptomycin (100 U/ml; Thermo Fisher Scientific, 15140-163). For selection of lentivirus-infected cells, puromycin was used at a concentration of 1.5 μg/ml, and hygromycin was used at a concentration of 250 μg/ml. Cells were tested for mycoplasma contamination using a MycoStrip detection kit (Invivogen, rep-mys-100).

### Lentivirus production

HEK293T cells were plated at a density of 100,000 cells/cm^2^ in 10- or 15-cm dishes 1 day before transfection. For 10-cm dishes, 10 μg of lentiviral transfer plasmid was combined with 8 μg of psPAX2 and 4 μg of pMD2.G in 250 μl of 300 mM NaCl. The DNA solution was then combined with 44 μg of polyethylenimine (PEI; Polysciences, 23966) in 250 μl of 300 mM NaCl for a 2:1 PEI:DNA ratio, vortexed, and incubated for 15 min at room temperature before addition to HEK293T cells. For 15-cm dishes, 20 μg of lentiviral transfer plasmid was combined with 16 μg of psPAX2 and 8 μg of pMD2.g in 500 μl of 300 mM NaCl. The DNA solution was then combined with 88 μg of PEI (2:1 PEI:DNA ratio) in 500 μl of 300 mM NaCl, vortexed, and incubated for 15 min at room temperature before addition to HEK293T cells. One day after transfection, growth medium was aspirated and replaced with fresh medium. Once per day for the following 2 days, medium containing lentiviral particles was collected and stored at 4°C. Lentivirus-containing medium was filtered through a 0.45-μm PES membrane and then combined with polyethylene glycol (PEG) precipitation solution [40% PEG 8000, 1.2 M NaCl in phosphate-buffered saline (PBS)] at a 3:1 ratio of medium: PEG precipitation solution. Samples were incubated at 4°C on a rocking platform overnight to precipitate lentiviral particles. The following day, samples were centrifuged at 1500*g* for 45 min at 4°C, after which, the supernatant was aspirated and the pellet dissolved in sterile Dulbecco's phosphate buffered saline (DPBS).

### Plasmids

sgRNA expression constructs were cloned into lentiCRISPR v2 using the Quick Ligation Kit (New England Biolabs, M2200L). Doxycycline-inducible expression constructs were cloned into pCW57-MCS1-P2A-MCS2 by digesting the plasmid with Nhe I–HF (New England Biolabs, R3131S) and Sal I–HF (New England Biolabs, R3138S) restriction enzymes and inserting synthetic DNA G-block fragments (Integrated DNA Technologies) using the NEBuilder HiFi DNA Assembly Master Mix (New England Biolabs, E2621L). pCW57-MCS1-P2A-MCS2 (Hygro) (Addgene, 80922) was a gift from A. Karpf (*40*). lentiCRISPR v2 (Addgene, 52961) was a gift from F. Zhang (*41*). pMD2.G (Addgene, 12259) and psPAX2 (Addgene 12260) were a gift from D. Trono. Human Brunello CRISPR knockout pooled library (Addgene, 73179) was a gift from D. Root and J. Doench (*42*). Plasmids were prepared from NEB Stable *E. coli* (New England Biolabs, C3040H) using Zymo Pure II plasmid prep kits (Zymo Research, D4203).

### CRISPR screening

The Brunello CRISPR library in LentiCRISPR V2 was amplified in Endura electrocompetent cells (Lucigen, 60242-1) according to the manufacturer’s recommendation. SYO-1 cells (1 × 10^7^ cells per 15-cm dish) were infected at a multiplicity of infection of approximately 0.5. Three replicates consisting of two 15-cm dishes (2 × 10^7^ cells per replicate) each were infected and maintained as three separate cultures throughout the experiment. One day after infection, medium was aspirated and replaced with fresh growth medium. Two days after infection, cells were trypsinized and replated (1.5 × 10^7^ cells per 15-cm dish) into growth medium containing puromycin (1.5 μg/ml). Cells were selected with puromycin for 9 days and then plated at a density of 1 × 10^7^ million cells per 15-cm dish, with six 15-cm dishes (three replicates of two plates each) for each treatment condition (DMSO or JQ1). The following day JQ1 (1 μM) was added to one set of plates and DMSO was added to the other. Medium containing JQ1 or DMSO was replaced every other day. DMSO-treated cells were passaged when they reached confluency which occurred every 2 to 3 days and replated at 1 × 10^7^ cells per 15-cm dish. After 9 days of selection with JQ1, cells were trypsinized and replated (1 × 10^6^ cells per 10-cm dish) and allowed to recover for 2 days without JQ1 selection after which JQ1 was added for seven more days. At this point, cells were collected by trypsinization and counted. The DMSO-treated samples yielded approximately 2 × 10^7^ cells per replicate, and genomic DNA was extracted from each replicate using a Quick-DNA Midiprep Plus Kit (Zymo, D4075). The JQ1-treated samples yielded approximately 3 × 10^5^ per replicate, and genomic DNA was extracted using the Quick-DNA Microprep Plus Kit (Zymo, D4074). Genomic DNA (up to 10 μg per 100-μl reaction) was amplified using Q5 Hot Start High-Fidelity 2X Master Mix according to the manufacturer’s protocol (New England Biolabs, M0494L). Polymerase chain reaction (PCR) cycling conditions were as follows: initial denaturation: 98°C (30 s); 28× cycles: 98°C (5 s), 63°C (20 s), and 72°C (30 s); final extension: 72°C (2 min). Following PCR amplification reactions corresponding to the same replicate were pooled together, and 50 μl was run into a 1% tris-acetate-EDTA agarose gel and purified using a QIAEX II Gel Extraction Kit (Qiagen, 20021). Libraries were pooled and sequenced on an Illumina NovaSeq instrument. MAGeCK (*43*) count and test commands were run using default parameters with nontargeting sgRNAs used for normalization.

### ChIP-seq

ChIP was performed with the buffer formulations and nomenclature as previously described (*44*). Typically 1 × 10^8^ to 2 × 10^8^ cells were cross-linked in suspension in growth medium at a concentration of 1 × 10^7^ cells/ml with 1% formaldehyde (MilliporeSigma, F8775) for 15 min at room temperature. Formaldehyde was quenched by addition of glycine to 0.125 M. Cross-linked cells were pelleted at 2000*g*, washed two times with PBS, snap frozen, and stored at −80°C before processing for ChIP. Cells were resuspended in lysis buffer 1 at a density of 1 × 10^7^ cells/ml and incubated for 10 min on ice. Nuclei were pelleted at 2000*g* and resuspended in lysis buffer 2 at a density of 1 × 10^7^ cells/ml and incubated for 10 min on ice. Nuclei were pelleted at 2000*g* and resuspended in lysis buffer 3 at a concentration of 5 × 10^7^ cells/ml. Nuclei suspensions were transferred to 1-ml milliTUBEs with AFA fibers (Covaris, 520130) and sonicated in a Covaris E220 focused ultrasonicator (duty factor, 10%; peak intensity pulse, 140; cycles per burst, 200; time, 500 s). Chromatin lysates were centrifuged at 20,000*g* to pellet insoluble material and quantified relative to a standard curve of bovine serum albumin dissolved in buffer 3 using a Bio-rad detergent compatible (DC) protein assay kit (Bio-Rad, 5000112). Samples were normalized to the same protein concentration (typically 1 to 2 mg/ml), and then Triton X-100 was added to a final concentration of 1%. Antibodies were added to the chromatin (table S2), and samples were incubated overnight at 4°C on an end-over-end rotator. The following day, 30 μl of Protein G Dynabead slurry (Thermo Fisher Scientific, 10003D) (prewashed with buffer 3 plus 1% Triton X-100) was added to each sample, and they were incubated for an addition 2 hours to allow for capture of immune complexes. Samples were washed four times for 5 min with 1 ml of wash buffer (radioimmunoprecipitation assay) in DynaMag magnet (Thermo Fisher Scientific, 12321D), twice with TE + NaCl and immune complexes were eluted by addition of 200 μl of Elution Buffer and incubation at 65°C for 2 hours. Dynabeads were cleared from the sample magnetically, and the supernatant containing the immunoprecipitated chromatin was supplemented with proteinase K (0.5 μg/ml) and incubated overnight at 65°C. The following day, DNA was purified using a ChIP DNA Clean and Concentrator kit (Zymo, D5205). ChIP-seq libraries were made using the KAPA HTP Biosystems Library Prep kit (Roche, KK8234) using PerkinElmer Unique Dual Index Barcodes for NovaSeq. ChIP-seq libraries were sequenced on an Illumina Nova-Seq instrument using single-end 50-cycle reads. Reads were aligned to the hg38 human genome assembly using Bowtie version 1.1.2 (*45*). Peaks were called using MACS2 (*46*), and occupancy plots were generated using DeepTools (*47*).

### RNA sequencing

RNA was extracted from cells using the RNeasy Mini Kit (Qiagen, 74106) using an on column DNaseI digestion step (Qiagen 79256). RNA was subjected to polyadenylated RNA enrichment using the NEBNext Poly (A) mRNA Magnetic Isolation Module Kit (New England Biolabs, E7490L), and libraries were synthesized using the NEBNext Ultra II Directional RNA Library Prep Kit for Illumina (New England Biolabs, E7760L). RNA-seq libraries were sequenced on an Illumina Nova-Seq instrument using single-end 50-cycle reads. Reads were aligned to the hg38 human genome assembly using Tophat version 2.1.0 (*48*) and quantified to the gene level with HTSeq version 0.6.0 (*49*). Batch correction was done using removal of unwanted variation from ribonucleic acid (RNA)–sequencing RUVseq (*50*), and differential gene expression analysis was performed using DESeq2 (*51*).

### Modified reduced representation bisulfite sequencing

Genomic DNA was isolated using the Qiagen AllPrep DNA/RNA Micro Kit and then prepared for reduced representation bisulfite sequencing as previously described (*52*–*56*). In short, approximately 200 ng of genomic DNA underwent restriction endonuclease digestion, size selection, bisulfite conversion, and index ligation to generate libraries for methylation sequencing. Unmethylated- λ-bacteriophage DNA (New England Biolabs) was included in each sample to calculate bisulfite conversion efficiency. On the basis of the frequency of unmethylated CpGs in λ-bacteriophage DNA, the estimated average bisulfate conversion efficiency is 99.05% ± 1.04% (SD). Sequencing was performed on an Illumina NextSeq 500 instrument using 75-bp single-end reads. DNA methylation analysis and quantification were performed using Trim Galore! v0.4.4, Bismark v0.16.3, and the Seqmonk bisulfite methylation pipeline as previously described (*52*, *53*, *57*). A custom reference genome for alignment was built on the basis of the T2T-CHM13 genome (*7*) to map KATNAL2 intron to ELOA3 genes in chromosome 18. For each sample, Bismark coverage files from ELOA3 regions were merged into a single file after methylation extraction for downstream methylation quantification.

### Drug dose-response experiments

One day before addition of drug, cells were plated in triplicate at a density of 40,000 cells per well in 24-well plates in 0.5 ml of growth medium. The following day, 0.5 ml of growth medium containing drug was added to the wells. Seventy-two hours after drug treatment, medium was carefully decanted from the plates, and cells were fixed using 1 ml of 4% formaldehyde (MilliporeSigma, F8775) in PBS (MilliporeSigma, P5358-10PAK) for 20 min at room temperature. After fixation, the PBS-formaldehyde solution was decanted, and plates were rinsed three times with cold tap water (Department of Water Management, Chicago, IL), and then air dried overnight. The following day, cells were stained with 1-ml crystal solution (MilliporeSigma, HT90132-1 L) for 1 hour after which the staining solution was decanted and plates were washed four times with cold tap water for 30 min. Plates were air dried overnight, and the following day crystal violet was dissolved in 0.5 ml of 10% acetic acid. The absorbance of 200 μl of sample was measured at 590 nm using a TECAN Infinite M1000 Pro plate reader. For each treatment, the absorbance values for drug treated wells were normalized to the average absorbance of the untreated wells.

### Phylogenetic analyses of human and primate long-read genome assemblies

The first copy of full-length ELOA3 unit sequence were retrieved from taking a start of a gene to a start of the next gene. With the full-length ELOA3 unit (4.6 to 5.9 kb), the loci of the consequent units were identified using BLAST with coverage threshold of 80%. Using the human T2T CHM13v2.0 assembly and six v.1.1 freeze primate assemblies of chimpanzee (PRJNA916736 and PRJNA916737), bonobo (PRJNA916735 and PRJNA916734), gorilla (PRJNA916732 and PRJNA916733), Bornean orangutan (PRJNA916742 and PRJNA916740), Sumatran orangutan (PRJNA916743 and PRJNA916741) and gibbon (PRJNA916729, PRJNA916728), the total of 230 full-length ELOA3 units were extracted and aligned with mafft (v7.487) (*58*). The maximum likelihood gene tree was reconstructed using iqtree (v2.1.2) (*59*) taking gibbon as the outgroup. “MFP” option was used to select the best model based on Bayesian information criterion as implemented in iqtree and bootstrap of 1000 was used to evaluate confidence of the tree topology. Least square dating approach implemented in iqtree was used for estimating the gene expansion time, based on the time calibrated with human-chimpanzee (6.2 Ma) and human-orangutan (15.2 Ma). The 95% confidence interval was computed based on 100 bootstrapping.

### Copy-number variability of ELOA3

Variability by species was quantified by making pairwise comparison among the ELOA3 repeat units within each species. The number of gap opening and mismatch per 1-kb sequence were counted for each pairwise comparison, and the significance of differences to human was tested using Wilcox nonparametric test.

### Pairwise dN/dS test

The 234 ELOA3 peptide sequences from the six ape species and human were aligned with mafft (v.7.489) (*58*), and the coding sequence (CDS) were retrieved from the aligned peptides using pal2nal (*60*). On the basis of the aligned CDS, pairwise dN/dS of human, chimpanzee, bonobo, gorilla, and B. and S. orangutans against gibbon were computed using PAML (v. 4.9j) (*61*).

### Examination of ELOA3 array in human genomes

The conservation of ELOA3 unit in human genome was examined from a set of 94 human haplotype assemblies generated by the HPRC. The location of the ELOA3 sequences were identified using BLAST with coverage threshold of 80%. The 23 haplotype assemblies that contain gaps 1 kb up or downstream of the ELOA3 array were filtered out, and the copy number of ELOA3 units in the remaining 71 assemblies were quantified. Conservation of the six human-specific amino acid substitutions in human were further examined using the 952 ELOA3 peptide sequences from the T2T assembly and 71 human haplotype assemblies.

### Analysis of the ELOA3 protein sequence

Phylogenetic analysis of the ELOA, ELOA2, and ELOA3 was performed using webPRANK (*62*), with default gap parameters and relaxed substitution scoring. For analysis of ELOA3 protein domains, proteins were aligned using Clustal Omega (*63*) and disordered regions were predicted using IUPred2A (*64*).

### Immunoprecipitation

SYO-1 cell pellets were resuspended in five cell pellet volumes of lysis buffer [10 mM Hepes-KOH (pH 7.9), 10 mM KCl, 1.5 mM MgCl_2_, 340 mM sucrose, 10% glycerol, and 0.2% Triton X-100] supplemented with protease inhibitors (MilliporeSigma, P8340) and phosphatase inhibitors (Thermo Fisher Scientific, A32957) and incubated on ice for 5 min. Nuclei were pelleted at by centrifugation at 2000*g* at 4°C for 5 min; supernatant was discarded and nuclei were then resuspended in five cell pellet volumes of lysis buffer. Benzonase (MilliporeSigma, E1014-25KU) (650 U/ml) was added, and DNA was digested on ice for 20 min. Benzonase digestion was stopped by the addition of 5 mM EDTA, 5 mM EGTA,, and 250 mM NaCl. Lysates were cleared by centrifugation at 20,000*g* at 4°C for 20 min and clarified lysates were incubated with anti-Flag agarose beads (MilliporeSigma, A2220-5ML) (50 μl of slurry per milliliter of sample) at 4°C for 1 hour. Beads were then washed four times at 4°C for 5 min with wash buffer [10 mM Hepes-KOH (pH 7.9), 10 mM KCl, 1.5 mM MgCl_2_, 340 mM sucrose, 10% glycerol, 0.2% Triton X-100, 5 mM EDTA, 5 mM EGTA, and 250 mM NaCl] using a volume equivalent to the lysate volume for each wash. Beads were washed once with 500 μl of 10 mM Hepes-KOH (pH 7.9) and 250 mM NaCl, and immune complexes were eluted with 200 μl of 10 mM Hepes-KOH (pH 7.9), 250 mM NaCl, 0.5% SDS, and Flag peptide (200 μg/ml; MilliporeSigma, F3290-4MG).

### Immunofluorescence

Coverslips were submerged in 70% ethanol, placed into six-well plates and then coated with 0.0004% poly-l-ornithine (MilliporeSigma, P4957-50ML) for 10 min and rinsed once with sterile water before plating cells. Cells were rinsed once with PBS and fixed with 4% formaldehyde in PBS for 10 min at room temperature. Cells were then rinsed once in PBS and permeabilized in TBS-TX [50 mM tris (pH 7.4), 200 mM NaCl, 0.25% Triton X-100, and 0.25% Tween] for 10 min at 4°C. Cells were then blocked by incubation in TBS-TX containing 5% BSA for 1 hour. Primary antibody dilutions were made in TBS-TX containing 5% BSA and 50-μl spots were placed onto a piece of parafilm. Coverslips were placed onto antibody solution cells facing down and incubated at 4°C overnight in a box lined with damp tissue paper for humidity. The following day, coverslips were transferred to six-well plates and washed four times with 2 ml of TBS-TX for 10 min. Secondary antibodies were diluted in TBS-TX supplemented with 4′,6-diamidino-2-Phenylindole, Dihydrochloride (DAPI; 2.5 μg/ml) (Thermo Fisher Scientific, D1306) and 0.5 ml of antibody solution was added to each coverslip in a six-well plate and incubated for 1 hour on a rapidly rocking platform protected from light. Coverslips were then washed four times with 2 ml of TBS-TX for 10 min and then mounted onto slides using Aqua-Mount (VWR, 41799-008). Fluorescence images were captured using a Nikon Eclipse Ts2R microscope equipped with a Nikon DS-Qi2 camera.

### Mass spectrometry

Mass spectrometry sample processing and analysis was performed at the University of Arkansas for Medical Science IDeA National Resource for Quantitative Proteomics as described (*65*, *66*) with the modification that protein reduction, alkylation and trypsin digestion were performed in solution.

### Immunoblotting

Protein samples were resolved on 4 to 20% acrylamide gels (Bio-Rad, 4561096) in running buffer (25 mM tris, 191 mM glycine, and 0.1% SDS) at a constant current of 20 mA per gel for 65 min. Proteins were transferred to polyvinylidene membranes (MilliporeSigma, IPVH00010) in transfer buffer (50 mM tris, 40 mM Glycine, 0.005% SDS, 20% methanol) for 90 min at 100 V. Blots were blocked with TBSTM [50 mM tris (pH 7.5), 200 mM NaCl, 0.1% Tween 20, and 5% nonfat dry milk] for 1 hour at room temperature. Antibodies were then diluted in TBSTM and incubated at 4°C overnight. The following day blots were washed three times for 10 min with TBST [50 mM tris (pH 7.5), 200 mM NaCl, 0.1% Tween 20], and secondary antibodies were diluted in TBST and incubated for 1 hour at room temperature. Blots were washed three times for 10 min with TBST [50 mM tris (pH 7.5), 200 mM NaCl, and 0.1% Tween 20] and incubated with Immobilon Crescendo Western HRP substrate (MilliporeSigma, WBLUR0500). Signal was visualized using a Chemidoc Touch imaging system (Bio-Rad).
