# Supplementary material for: ELOA3: A primate-specific RNA polymerase II elongation factor encoded by a tandem repeat gene cluster
Source: Sci Adv. 2023 Nov 22;9(47):eadj1261. doi: 10.1126/sciadv.adj1261 (PMC10664989; doi:10.1126/sciadv.adj1261)
Supplement: Supplementary file 1 — Figs. S1 to S4 Tables S1 to S3 [file sciadv.adj1261_sm.pdf]

Science Advances
AAAS

Supplementary Materials for

ELOA3: A primate-specific RNA polymerase II elongation factor encoded by a tandem repeat gene cluster

Marc A. J. Morgan *et al.*

Corresponding author: Ali Shilatifard, ash@northwestern.edu

Sci. Adv. **9**, eadj1261 (2023)
DOI: 10.1126/sciadv.adj1261

This PDF file includes:

Figs. S1 to S4
Tables S1 to S3

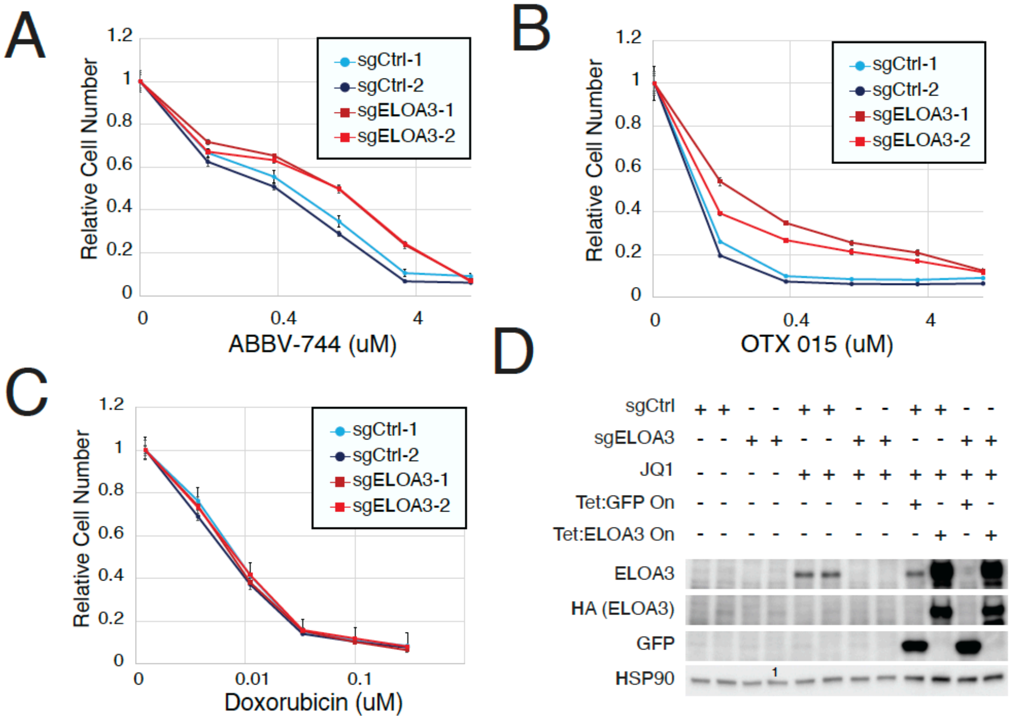

Supplemental Figure S1. ELOA3 increases BETi sensitivity in SYO-1 cells

(A-C) Drug dose response experiments performed in control or *ELOA3* sgRNA infected SYO-1 cells treated with (A) ABBV-744, (B) OTX 015 or (C) doxorubicin. Data represent mean ± SD, n = 3. (D) Immunoblotting of whole cell extracts from control and ELOA3 sgRNA treated cells and expressing Tet:GFP or Tet:ELOA3 constructs and treated with DMSO or JQ1 (5uM) for 24 hours.

A

Species	ELOA3 gene copy number (# of CDS/#of full-length gene)	
	Hap 1	Hap 2
Human (CHM13v2.0)	12 (11)	n/a
Chimpanzee	55 (54)	50 (49)
Bonobo	31 (30)	30 (29)
Gorilla	22 (21)	23 (22)
B. Orangutan	39 (39)	36 (36)
S. Orangutan	43 (44)	67 (67)
Gibbon	32 (31)	36 (32)
Sum	234 (230)	242 (235)

B

Group		N	ELOA3 copy number			
			Mean	SD	Median	Mode
All		71	13.2	5.53	14	14
Sex	Female	43	13.9	5.77	14	14
	Male	28	12.3	5.07	13	14
Population	AFR	37	14.4	5.57	14	14
	AMR	22	11.0	4.89	10.5	15
	EAS	8	12.8	6.76	13.5	8
	EUR	2	16.0	2.83	16	16
	SAS	2	16.0	1.41	16	16

C

Position in MSA	Position in human	Human	Chimpanzee	Bonobo	Gorilla	B. orangutan	S. orangutan	Gibbon
12	12	G (949)	E (55)	E/K (1/30)	E (22)	Q (39)	Q (43)	E (32)
23	23	E (948)	D (55)	D (31)	D (22)	N (39)	N (43)	D (32)
334	303	A/T (436/501)	T (52)	T (30)	T (22)	T (39)	T (43)	T (32)
413	383	A (936)	P (52)	P (30)	P (22)	H (39)	H (43)	H (32)
500	470	A/P (886/21)	A (52)	A (30)	A (21)	A (39)	A (43)	A (32)
597	512	R (895)	Q (52)	Q (30)	E (21)	E (39)	E (43)	E (31)

D

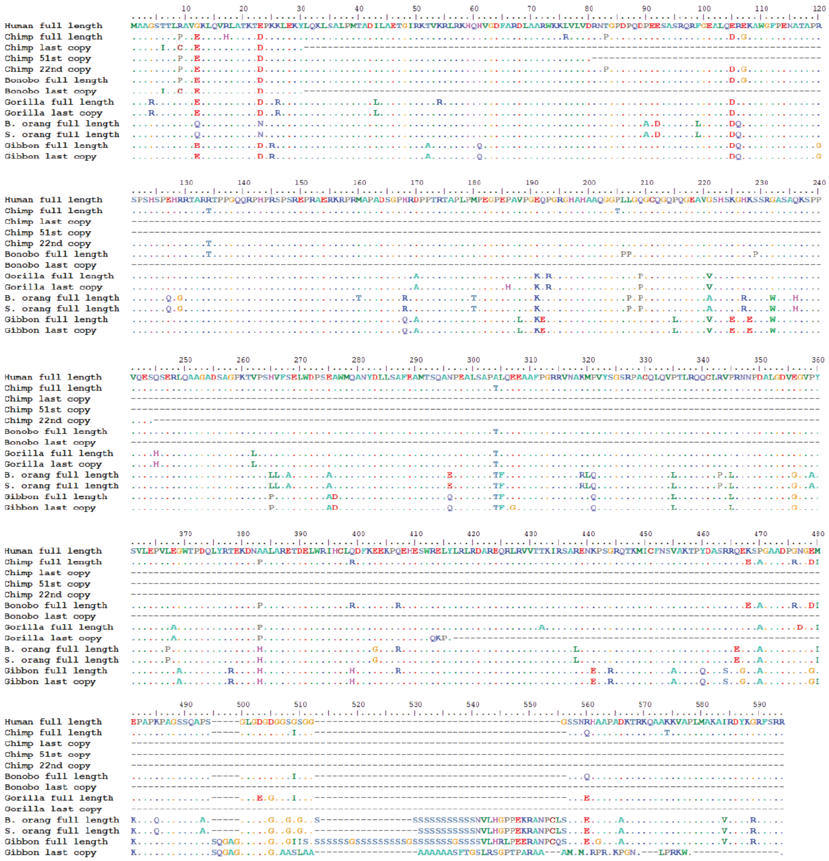

Supplemental Figure S2. Copy number and protein coding sequence analysis of human and primate ELOA3 genes.

(A) Table of the copy number of ELOA3 genes within the gene cluster in the human CHM13 genome compared to 7 primate genomes. (B) Table of ELOA3 gene copy number across subsets of the human population. AFR: African, AMR: Admixed American, EAS: East Asian, EUR: European, SAS: South Asian. (C) Table of the 5 human-specific amino acid substitutions in the ELOA3 protein coding sequence as compared to primate species. Numbers beneath amino acid symbols indicate the number of ELOA3 genes that contain a given amino acid. MSA: multiple sequence alignment. (D) Multiple sequence alignment of human and primate ELOA3 coding sequences. Positions that differ from the human sequence are indicated as colored amino acid symbols, dots indicate identical residues and dashes indicate alignment gaps.

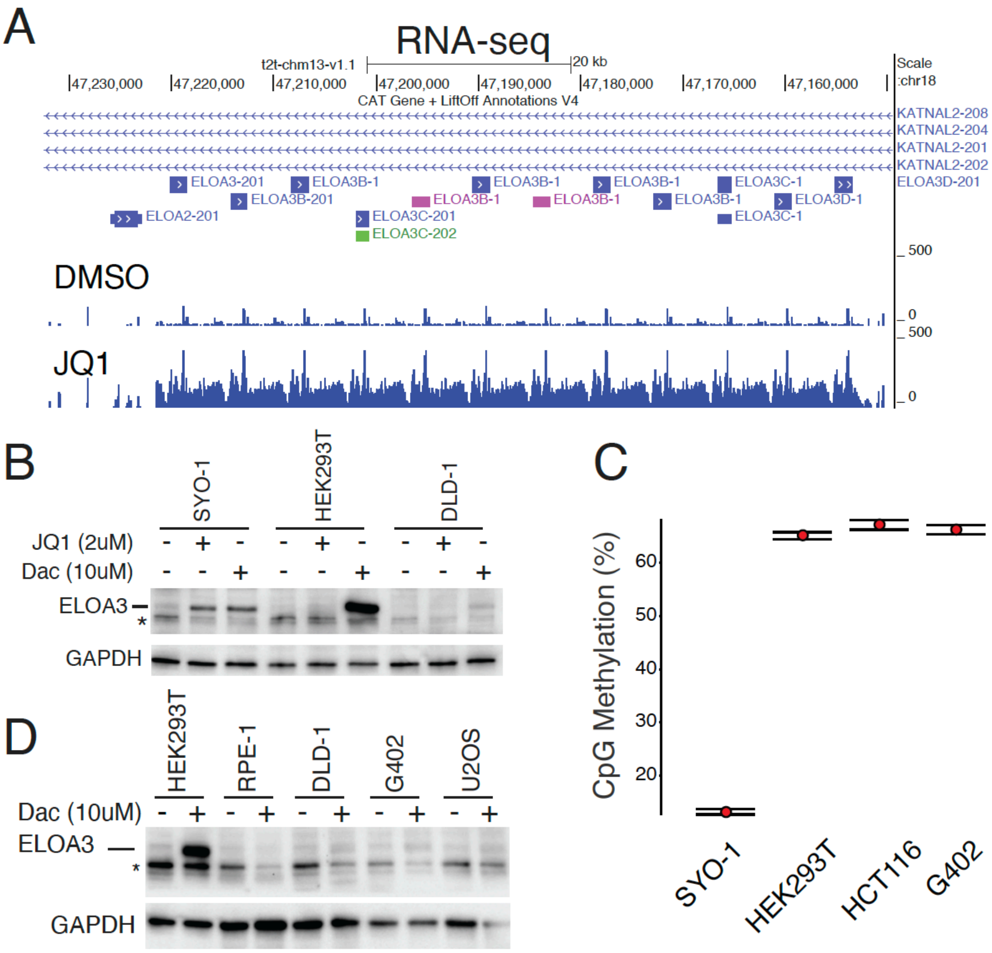

Supplemental Figure S3. Regulation of the ELOA3 locus by JQ1 treatment and DNA methyltransferase inhibition

(A) RNA sequencing data from DMSO and JQ1 treated SYO-1 cells aligned to the T2T chm13-v1.1 genome assembly, allowing for multiple alignments (Nmax=100). (B) Western blots of whole cell extracts from cells treated with JQ1 or decitabine. Asterisk indicates a non-specific band observed in several cell lines. (C) Plot of reduced representation bisulfite sequencing (RRBS) data displaying the percentage of methylated CpGs at the *ELOA3* gene cluster in the cell lines indicated. Error bars represent standard error. (D) Western blots of whole cell extracts from cells treated with decitabine. Asterisk indicates a non-specific band observed in several cell lines.

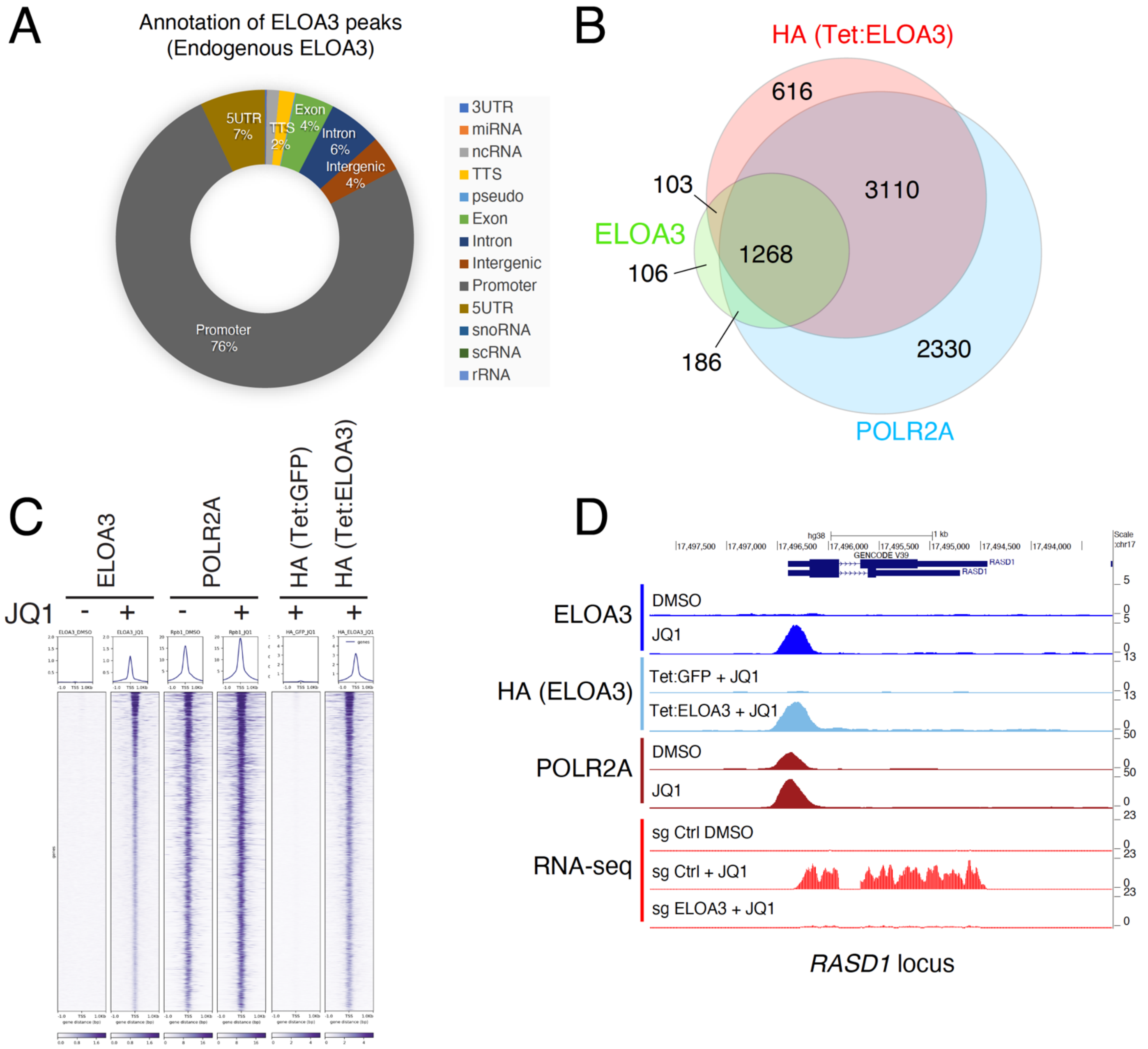

Supplemental Figure S4. ELOA3 ChIP-seq analysis
(A) HOMER analysis of the distribution of ELOA3 peaks genome-wide. (B) Venn diagram displaying the overlap between endogenous ELOA3 peaks (green), HA-tagged ELOA3 peaks (red) and RNAPII (blue). (C) ChIP-seq occupancy plots for the antibodies indicated centered on all ELOA3 peaks detected in SYO-1 cells. (D) UCSC genome browser displaying ChIP-seq and RNA-seq tracks from SYO-1 cells treated with DMSO or JQ1 using the antibodies indicated.

Oligonucleotide Name	Sequence
LentiCRISPR V2 Forward Stagger 1	AATGATACGGCGACCACCGAGATCTACACACTGCATAACACTCTTTCCCTACACGACGCTCTTCCGATCTTTGTGGAAAGGACGAAACACCG
LentiCRISPR V2 Forward Stagger 2	AATGATACGGCGACCACCGAGATCTACACACTGCATAACACTCTTTCCCTACACGACGCTCTTCCGATCTCTTGTGGAAAGGACGAAACACCG
LentiCRISPR V2 Forward Stagger 3	AATGATACGGCGACCACCGAGATCTACACACTGCATAACACTCTTTCCCTACACGACGCTCTTCCGATCTGCTTGTGGAAAGGACGAAACACCG
LentiCRISPR V2 Forward Stagger 4	AATGATACGGCGACCACCGAGATCTACACACTGCATAACACTCTTTCCCTACACGACGCTCTTCCGATCTAGCTTGTGGAAAGGACGAAACACCG
LentiCRISPR V2 Forward Stagger 5	AATGATACGGCGACCACCGAGATCTACACACTGCATAACACTCTTTCCCTACACGACGCTCTTCCGATCTCAACTTGTGGAAAGGACGAAACACCG
LentiCRISPR V2 Forward Stagger 6	AATGATACGGCGACCACCGAGATCTACACACTGCATAACACTCTTTCCCTACACGACGCTCTTCCGATCTTGCACTTGTGGAAAGGACGAAACACCG
LentiCRISPR V2 Forward Stagger 7	AATGATACGGCGACCACCGAGATCTACACACTGCATAACACTCTTTCCCTACACGACGCTCTTCCGATCTACGCAATTGTGGAAAGGACGAAACACCG
LentiCRISPR V2 Forward Stagger 8	AATGATACGGCGACCACCGAGATCTACACACTGCATAACACTCTTTCCCTACACGACGCTCTTCCGATCTGAAGACCTTGTGGAAAGGACGAAACACCG
LentiCRISPR V2 Reverse i7 index 1	CAAGCAGAAGACGGCATACGAGATCGGTTCAAGTGACTGGAGTTCAGACGTGTGCTCTTCCGATCTCCAATTCCCACTCCTTTCAAGACCT
LentiCRISPR V2 Reverse i7 index 2	CAAGCAGAAGACGGCATACGAGATGCTGGATTGTGACTGGAGTTCAGACGTGTGCTCTTCCGATCTCCAATTCCCACTCCTTTCAAGACCT
LentiCRISPR V2 Reverse i7 index 3	CAAGCAGAAGACGGCATACGAGATTAACTCGGGTGACTGGAGTTCAGACGTGTGCTCTTCCGATCTCCAATTCCCACTCCTTTCAAGACCT
LentiCRISPR V2 Reverse i7 index 4	CAAGCAGAAGACGGCATACGAGATATTGGAGGGTGACTGGAGTTCAGACGTGTGCTCTTCCGATCTCCAATTCCCACTCCTTTCAAGACCT
LentiCRISPR V2 Reverse i7 index 5	CAAGCAGAAGACGGCATACGAGATTAGTCTAAGTGACTGGAGTTCAGACGTGTGCTCTTCCGATCTCCAATTCCCACTCCTTTCAAGACCT
LentiCRISPR V2 Reverse i7 index 6	CAAGCAGAAGACGGCATACGAGATCGGTGACCGTGACTGGAGTTCAGACGTGTGCTCTTCCGATCTCCAATTCCCACTCCTTTCAAGACCT
ELOA3 sgRNA 1 sense	CACC G CATGACCGCAGACATCCTGG
ELOA3 sgRNA 1 antisense	AAAC CCAGGATGTCTGCGGTCATGC
ELOA3 sgRNA 2 sense	CACC GCCTGACCCGCAGGACCCTG
ELOA3 sgRNA 2 antisense	AAAC CAGGGTCCTGCGGGTCAGGC
Non-targeting sgRNA 1 sense	CACC GTAAGATGATAAGGCGTATG
Non-targeting sgRNA 1 antisense	AAAC CATACGCCTTATCATCTTAC
Non-targeting sgRNA 2 sense	CACC G AAGAGCGAATCGATTTCGTG
Non-targeting sgRNA 2 antisense	AAAC CACGAAATCGATTCGCTCTTC
gBlock name	Sequence
Flag-2xHA-ELOA3	agtgaaccgtcagatcgcctggagaattggctagcACCATGGATTACAAGGATGACGATGACAAGGGTGGCtacccctacgacgtgcccgactacgccggctatccgtatgatgtcccggactatgcaGCTGCGGGCTCAACTACTCTTCGTGCTGTGGGCAAACTCCAAGTCAGACTCGCAACCAAAACAGAACCCAAGAAACTCGAAAAGTACCTGCAAAAGCTGTCAGCTCTGCCAATGACAGCCGATATTTTGGCTGAAACCGGGATTAGGAAAACAGTAAAACGTTTGAGGAAACATCAACATGTTGGAGATTTCGCTCGGGATCTGGCTGCGCGATGGAAGAAACTTGTACTGGTCGATCGGAATACAGGTCCCGATCCTCAAGATCCCGAAGAATCAGCAAGCAGGCAACGATTTGGAGAAGCCCTGCAAGAAAGAGAGAAAGCATGGGGATTTCCCGAGAATGCAACTGCTCCTCGGAGTCCCAGCCATTCTCCAGAACATCGCAGAACCGCGAGAAGGACCCCGCCAGGTCAGCAACGGCCCCATCCCAGAAGCCCATCTAGGGAACCAAGGGCTGAACGCAAACGACCACGAATGGCTCCTGCAGACAGTGGGCCACATAGAGATCCACCCACACGAACGGCCCCTCTGCCCATGCCTGAAGGTCCAGAACCTGCCGTCCCAGGCGAACAGCCGGGACGGGGACATGCACATGCAGCCCAAGGCGGACCATTACTCGGACAGGGATGTCAAGGGCAGCCTCAAGGTGAGGCCGTAGGCTCCCATTCCAAAGGACATAAGAGTAGCAGAGGAGCATCAGCACAAAAGAGTCCCCCAGTTCAAGAGTCTCAAAGTGAAAGATTGCAAGCCGCAGTGGCAGACAGCGCAGGCCCCAAGACCGTTCCTTCTCACGTGTTTTCTGAACTGTGGGATCCAAGCGAAGCGTGGATGCAAGCGAATTATGACTTACTCAGCGCCTTCGAAGCTATGACTAGTCAAGCCAATCCGGAAGCCCTTAGCGCCCCCACCCTTCAAGAAGAGGCAGCCTTTCCAGGCCGTCGGGTCAATGCCAAAATGCCAGTCTATAGCGGGAGCCGGCCAGCATGTCAATTGCAAGTACCCACATTGCGTCAACAATGTCTGCGCGTTCCCCGCAATAACCCCGATGCTTTAGGGGATGTTGAGGGCGTGCCATATTCTGCCTTAGAGCCGGTGTTAGAGGGATGGACACCAGACCAACCCTATAGAACGGAAAAGGATAACGCTGCTTTAGCACGGGAAACGGACGAGCTGTGGAGAATCCACTGTCTTCAAGATTTTAAAGAGGAGAAACCGCAAGAACATGAAAGCTGGAGAGAATTATATTTACGCCTGCGCGATGCACGCGAACAACGCTTGCGGGTGGTCACTACCAAGATTAGGTCTGCTAGGGAGAATAAGCCTTCCGGGCGTCAAACCAAAATGATTTGCTTTAATTCCGTCGCTAAAACACCCTACGACGCCTCTAGACGCCAGGAAAAGAGTGCCGGTGCGGCAGATCCTGGGAACGGCGAAATGGAACCTGCGCCAAAACCTGCCGGGTCATCCCAAGCACCATCAGGGCTGGGCGATGGTGATGGTGGGTCTGTTTCCGGTGGTGGTAGTAGTAATCGACATGCCGCTCCAGCCGATAAGACTAGGAAGCAAGCAGCGAAGAAGGTCGCACCTCTTATGGCAAAAGCCATCCGGGATTATAAAGGCCGGTTTAGCAGAAGGTAGgtcgacaccggttccggagccacgaacttctctct
Flag-2xHA-ELOA3 ΔTFIIS	agtgaaccgtcagatcgcctggagaattggctagcACCATGGATTACAAGGATGACGATGACAAGGGTGGCtacccctacgacgtgcccgactacgccggctatccgtatgatgtcccggactatgcaCCCGATCCTCAAGATCCCGAAGAATCAGCAAGCAGGCAACGATTTGGAGAAGCCCTGCAAGAAAGAGAGAAAGCATGGGGATTTCCCGAGAATGCAACTGCTCCTCGGAGTCCCAGCCATTCTCCAGAACATCGCAGAACCGCGAGAAGGACCCCGCCAGGTCAGCAACGGCCCCATCCCAGAAGCCCATCTAGGGAACCAAGGGCTGAACGCAAACGACCACGAATGGCTCCTGCAGACAGTGGGCCACATAGAGATCCACCCACACGAACGGCCCCTCTGCCCATGCCTGAAGGTCCAGAACCTGCCGTCCCAGGCGAACAGCCGGGACGGGGACATGCACATGCAGCCCAAGGCGGACCATTACTCGGACAGGGATGTCAAGGGCAGCCTCAAGGTGAGGCCGTAGGCTCCCATTCCAAAGGACATAAGAGTAGCAGAGGAGCATCAGCACAAAAGAGTCCCCCAGTTCAAGAGTCTCAAAGTGAAAGATTGCAAGCCGCAGTGGCAGACAGCGCAGGCCCCAAGACCGTTCCTTCTCACGTGTTTTCTGAACTGTGGGATCCAAGCGAAGCGTGGATGCAAGCGAATTATGACTTACTCAGCGCCTTCGAAGCTATGACTAGTCAAGCCAATCCGGAAGCCCTTAGCGCCCCCACCCTTCAAGAAGAGGCAGCCTTTCCAGGCCGTCGGGTCAATGCCAAAATGCCAGTCTATAGCGGGAGCCGGCCAGCATGTCAATTGCAAGTACCCACATTGCGTCAACAATGTCTGCGCGTTCCCCGCAATAACCCCGATGCTTTAGGGGATGTTGAGGGCGTGCCATATTCTGCCTTAGAGCCGGTGTTAGAGGGATGGACACCAGACCAACCCTATAGAACGGAAAAGGATAACGCTGCTTTAGCACGGGAAACGGACGAGCTGTGGAGAATCCACTGTCTTCAAGATTTTAAAGAGGAGAAACCGCAAGAACATGAAAGCTGGAGAGAATTATATTTACGCCTGCGCGATGCACGCGAACAACGCTTGCGGGTGGTCACTACCAAGATTAGGTCTGCTAGGGAGAATAAGCCTTCCGGGCGTCAAACCAAAATGATTTGCTTTAATTCCGTCGCTAAAACACCCTACGACGCCTCTAGACGCCAGGAAAAGAGTGCCGGTGCGGCAGATCCTGGGAACGGCGAAATGGAACCTGCGCCAAAACCTGCCGGGTCATCCCAAGCACCATCAGGGCTGGGCGATGGTGATGGTGGGTCTGTTTCCGGTGGTGGTAGTAGTAATCGACATGCCGCTCCAGCCGATAAGACTAGGAAGCAAGCAGCGAAGAAGGTCGCACCTCTTATGGCAAAAGCCATCCGGGATTATAAAGGCCGGTTTAGCAGAAGGTAGgtcgacaccggttccggagccacgaacttctctct
Flag-2xHA-ELOA3 ΔDD1	agtgaaccgtcagatcgcctggagaattggctagcACCATGGATTACAAGGATGACGATGACAAGGGTGGCtacccctacgacgtgcccgactacgccggctatccgtatgatgtcccggactatgcaGCTGCGGGCTCAACTACTCTTCGTGCTGTGGGCAAACTCCAAGTCAGACTCGCAACCAAAACAGAACCCAAGAAACTCGAAAAGTACCTGCAAAAGCTGTCAGCTCTGCCAATGACAGCCGATATTTTGGCTGAAACCGGGATTAGGAAAACAGTAAAACGTTTGAGGAAACATCAACATGTTGGAGATTTCGCTCGGGATCTGGCTGCGCGATGGAAGAAACTTGTACTGGTCGATCGGAATACAGGTCCCACATTGCGTCAACAATGTCTGCGCGTTCCCCGCAATAACCCCGATGCTTTAGGGGATGTTGAGGGCGTGCCATATTCTGCCTTAGAGCCGGTGTTAGAGGGATGGACACCAGACCAACCCTATAGAACGGAAAAGGATAACGCTGCTTTAGCACGGGAAACGGACGAGCTGTGGAGAATCCACTGTCTTCAAGATTTTAAAGAGGAGAAACCGCAAGAACATGAAAGCTGGAGAGAATTATATTTACGCCTGCGCGATGCACGCGAACAACGCTTGCGGGTGGTCACTACCAAGATTAGGTCTGCTAGGGAGAATAAGCCTTCCGGGCGTCAAACCAAAATGATTTGCTTTAATTCCGTCGCTAAAACACCCTACGACGCCTCTAGACGCCAGGAAAAGAGTGCCGGTGCGGCAGATCCTGGGAACGGCGAAATGGAACCTGCGCCAAAACCTGCCGGGTCATCCCAAGCACCATCAGGGCTGGGCGATGGTGATGGTGGGTCTGTTTCCGGTGGTGGTAGTAGTAATCGACATGCCGCTCCAGCCGATAAGACTAGGAAGCAAGCAGCGAAGAAGGTCGCACCTCTTATGGCAAAAGCCATCCGGGATTATAAAGGCCGGTTTAGCAGAAGGTAGgtcgacaccggttccggagccacgaacttctctct
Flag-2xHA-ELOA3 ΔELOA	agtgaaccgtcagatcgcctggagaattggctagcACCATGGATTACAAGGATGACGATGACAAGGGTGGCtacccctacgacgtgcccgactacgccggctatccgtatgatgtcccggactatgcaGCTGCGGGCTCAACTACTCTTCGTGCTGTGGGCAAACTCCAAGTCAGACTCGCAACCAAAACAGAACCCAAGAAACTCGAAAAGTACCTGCAAAAGCTGTCAGCTCTGCCAATGACAGCCGATATTTTGGCTGAAACCGGGATTAGGAAAACAGTAAAACGTTTGAGGAAACATCAACATGTTGGAGATTTCGCTCGGGATCTGGCTGCGCGATGGAAGAAACTTGTACTGGTCGATCGGAATACAGGTCCCGATCCTCAAGATCCCGAAGAATCAGCAAGCAGGCAACGATTTGGAGAAGCCCTGCAAGAAAGAGAGAAAGCATGGGGATTTCCCGAGAATGCAACTGCTCCTCGGAGTCCCAGCCATTCTCCAGAACATCGCAGAACCGCGAGAAGGACCCCGCCAGGTCAGCAACGGCCCCATCCCAGAAGCCCATCTAGGGAACCAAGGGCTGAACGCAAACGACCACGAATGGCTCCTGCAGACAGTGGGCCACATAGAGATCCACCCACACGAACGGCCCCTCTGCCCATGCCTGAAGGTCCAGAACCTGCCGTCCCAGGCGAACAGCCGGGACGGGGACATGCACATGCAGCCCAAGGCGGACCATTACTCGGACAGGGATGTCAAGGGCAGCCTCAAGGTGAGGCCGTAGGCTCCCATTCCAAAGGACATAAGAGTAGCAGAGGAGCATCAGCACAAAAGAGTCCCCCAGTTCAAGAGTCTCAAAGTGAAAGATTGCAAGCCGCAGTGGCAGACAGCGCAGGCCCCAAGACCGTTCCTTCTCACGTGTTTTCTGAACTGTGGGATCCAAGCGAAGCGTGGATGCAAGCGAATTATGACTTACTCAGCGCCTTCGAAGCTATGACTAGTCAAGCCAATCCGGAAGCCCTTAGCGCCCCCACCCTTCAAGAAGAGGCAGCCTTTCCAGGCCGTCGGGTCAATGCCAAAATGCCAGTCTATAGCGGGAGCCGGCCAGCATGTCAATTGCAAGTACCTTCCGGGCGTCAAACCAAAATGATTTGCTTTAATTCCGTCGCTAAAACACCCTACGACGCCTCTAGACGCCAGGAAAAGAGTGCCGGTGCGGCAGATCCTGGGAACGGCGAAATGGAACCTGCGCCAAAACCTGCCGGGTCATCCCAAGCACCATCAGGGCTGGGCGATGGTGATGGTGGGTCTGTTTCCGGTGGTGGTAGTAGTAATCGACATGCCGCTCCAGCCGATAAGACTAGGAAGCAAGCAGCGAAGAAGGTCGCACCTCTTATGGCAAAAGCCATCCGGGATTATAAAGGCCGGTTTAGCAGAAGGTAGgtcgacaccggttccggagccacgaacttctctct
Flag-2xHA-ELOA3 ΔDD2	agtgaaccgtcagatcgcctggagaattggctagcACCATGGATTACAAGGATGACGATGACAAGGGTGGCtacccctacgacgtgcccgactacgccggctatccgtatgatgtcccggactatgcaGCTGCGGGCTCAACTACTCTTCGTGCTGTGGGCAAACTCCAAGTCAGACTCGCAACCAAAACAGAACCCAAGAAACTCGAAAAGTACCTGCAAAAGCTGTCAGCTCTGCCAATGACAGCCGATATTTTGGCTGAAACCGGGATTAGGAAAACAGTAAAACGTTTGAGGAAACATCAACATGTTGGAGATTTCGCTCGGGATCTGGCTGCGCGATGGAAGAAACTTGTACTGGTCGATCGGAATACAGGTCCCGATCCTCAAGATCCCGAAGAATCAGCAAGCAGGCAACGATTTGGAGAAGCCCTGCAAGAAAGAGAGAAAGCATGGGGATTTCCCGAGAATGCAACTGCTCCTCGGAGTCCCAGCCATTCTCCAGAACATCGCAGAACCGCGAGAAGGACCCCGCCAGGTCAGCAACGGCCCCATCCCAGAAGCCCATCTAGGGAACCAAGGGCTGAACGCAAACGACCACGAATGGCTCCTGCAGACAGTGGGCCACATAGAGATCCACCCACACGAACGGCCCCTCTGCCCATGCCTGAAGGTCCAGAACCTGCCGTCCCAGGCGAACAGCCGGGACGGGGACATGCACATGCAGCCCAAGGCGGACCATTACTCGGACAGGGATGTCAAGGGCAGCCTCAAGGTGAGGCCGTAGGCTCCCATTCCAAAGGACATAAGAGTAGCAGAGGAGCATCAGCACAAAAGAGTCCCCCAGTTCAAGAGTCTCAAAGTGAAAGATTGCAAGCCGCAGTGGCAGACAGCGCAGGCCCCAAGACCGTTCCTTCTCACGTGTTTTCTGAACTGTGGGATCCAAGCGAAGCGTGGATGCAAGCGAATTATGACTTACTCAGCGCCTTCGAAGCTATGACTAGTCAAGCCAATCCGGAAGCCCTTAGCGCCCCCACCCTTCAAGAAGAGGCAGCCTTTCCAGGCCGTCGGGTCAATGCCAAAATGCCAGTCTATAGCGGGAGCCGGCCAGCATGTCAATTGCAAGTACCCACATTGCGTCAACAATGTCTGCGCGTTCCCCGCAATAACCCCGATGCTTTAGGGGATGTTGAGGGCGTGCCATATTCTGCCTTAGAGCCGGTGTTAGAGGGATGGACACCAGACCAACCCTATAGAACGGAAAAGGATAACGCTGCTTTAGCACGGGAAACGGACGAGCTGTGGAGAATCCACTGTCTTCAAGATTTTAAAGAGGAGAAACCGCAAGAACATGAAAGCTGGAGAGAATTATATTTACGCCTGCGCGATGCACGCGAACAACGCTTGCGGGTGGTCACTACCAAGATTAGGTCTGCTAGGGAGAATAAGTAGgtcgacaccggttccggagccacgaacttctctct
Flag-2xHA-ELOA	agtgaaccgtcagatcgcctggagaattggctagcACCATGGATTACAAGGATGACGATGACAAGGGTGGCtacccctacgacgtgcccgactacgccggctatccgtatgatgtcccggactatgcaGCAGCCGAATCCGCTCTGCAGGTCGTTGAAAAGCTCCAAGCACGGTTGGCTGCCAATCCCGATCCAAAGAAATTATTAAAGTACCTCAAGAAGCTTTCAACTTTGCCAATAACTGTGGATATCCTGGCAGAAACGGGTGTGGGCAAGACCGTGAACTCCCTGAGGAAGCATGAACACGTCGGTTCCTTCGCAAGAGATTTGGTCGCGCAATGGAAGAAACTCGTGCCAGTAGAGCGGAACGCAGAACCGGACGAGCAAGATTTCGAAAAGTCCAACTCACGCAAAAGGCCGAGAGACGCACTTCAAAAGGAAGAAGAAATGGAAGGAGATTATCAGGAGACTTGGAAGGCTACAGGCTCTAGAAGTTACTCCCCGGATCATAGACAAAAGAAGCACAGAAAGCTGTCAGAACTGGAACGTCCACATAAGGTCTCCCATGGCCACGAACGGCGCGACGAACGGAAACGCTGCCATCGCATGAGCCCGACATATAGCTCCGATCCCGAATCCAGTGACTACGGACACGTACAGTCACCACCCAGTTGCACATCACCACACCAAATGTATGTGGATCATTATCGGAGTCTCGAAGAAGATCAAGAACCAATCGTGTCCCATCAAAAGCCAGGAAAGGGTCATAGTAACGCTTTCCAAGATCGGCTTGGTGCATCACAGGAGAGACATTTAGGGGAGCCTCACGGCAAGGGTGTGGTAAGCCAGAATAAAGAACATAAGTCCAGTCATAAAGATAAGAGACCAGTAGACGCAAAATCTGACGAAAAGGCTAGTGTCGTCTCACGGGAAAAGAGTCATAAAGCTCTGAGTAAGGAAGAAAATCGGAGACCGCCTAGTGGAGATAACGCGCGCGAAAAGCCCCCGAGTTCAGGAGTCAAGAAGGAGAAAGATCGCGAAGGGAGTTCATTAAAGAAGAAGTGCCTGCCACCAAGCGAAGCAGCATCTGATAATCATCTTAAGAAACCCAAACATCGCGATCCGGAAAAGGCGAAGTTAGATAAGTCTAAACAGGGGCTTGATTCCTTCGATACGGGGAAGGGCGCCGGCGATTTACTGCCAAAAGTGAAGGAGAAAGGCAGCAATAATCTGAAAACACCTGAGGGTAAGGTTAAGACCAACCTCGACCGCAAATCCCTCGGGTCACTGCCAAAGGTGGAAGAAACCGACATGGAAGACGAGTTTGAACAACCTACTATGTCCTTCGAGTCTTATCTTTCCTACGATCAACCTCGTAAGAAGAAGAAGAAAATCGTTAAGACCAGTGCAACCGCGCTGGGCGACAAGGGCCTCAAGAAGAACGATTCCAAGTCAACGGGAAAGAATCTGGATTCTGTGCAAAAGCTCCCTAAAGTCAATAAGACTAAAAGTGAGAAACCCGCGGGCGCCGACCTGGCAAAACTCCGCAAAGTCCCAGACGTTCTGCCCGTACTGCCTGATCTTCCATTGCCTGCAATCCAAGCAAACTATAGGCCGCTTCCCTCACTTGAACTCATTTCTTCATTTCAACCGAAAAGAAAGGCCTTTAGCTCTCCTCAAGAGGAAGAAGAGGCCGGGTTCACAGGAAGAAGGATGAACTCTAAAATGCAAGTTTACTCAGGATCAAAATGCGCTTACCTGCCAAAGATGATGACACTGCATCAACAGTGTATTAGAGTCTTGAAGAATAATATAGACTCTATTTTCGAGGTTGGCGGCGTGCCTTATTCCGTACTGGAGCCTGTCCTGGAAAGATGCACGCCAGACCAATTGTACAGGATTGAAGAGTATAACCACGTGCTCATCGAGGAGACGGACCAGCTGTGGAAGGTCCACTGCCATAGAGATTTCAAAGAGGAGCGACCAGAGGAATACGAATCTTGGCGGGAAATGTATCTTAGACTGCAAGATGCGCGGGAACAACGATTAAGGGTCCTTACCAAGAACATACAATTTGCCCACGCTAACAAACCAAAGGGGCGCCAGGCTAAAATGGCATTCGTAAATAGCGTAGCAAAACCACCACGGGATGTTAGGCGGCGACAAGAGAAATTCGGCACTGGCGGTGCGGCCGTGCCCGAAAAGATCAAAATAAAACCCGCGCCATATCCAATGGGTTCTAGTCACGCCAGTGCTTCCTCTATTTCCTTCAATCCAAGTCCGGAAGAACCCGCATACGACGGACCCAGCACTTCTTCCGCTCATCTGGCTCCCGTCGTTAGCTCCACCGTCTCATACGACCCACGGAAGCCAACCGTCAAGAAGATAGCTCCCATGATGGCTAAAACGATCAAGGCCTTTAAGAATAGGTTTAGCAGGCGGTGAgtcgacaccggttccggagccacgaacttctctct

Supplemental Table S1. Oligonucleotides and synthetic DNA

Name	Supplier	Catalog #	RRID	Lot #	Immunoblotting	ChIP	Immunofluorescence
ELOA3	Atlas Antibodies	HPA050086	AB_2681009	R60012	1:1,000	10ul / 1mg chromatin	1:100
HA-tag	Cell Signaling Technology	3724S	AB_1549585	Lot 10, 12/2024	1:2,000	10ul / 1mg chromatin	1:100
GFP	Santa Cruz	sc-9996	AB_627695	B2521	1:1,000	N/A	N/A
HSP90	Santa Cruz	sc-13119	AB_675659	A2020	1:10,000	N/A	N/A
POLR2A (Rpb1)	Cell Signaling Technology	14958S	AB_2687876	Lot 4, 12/2023	1:1,000	10ul / 1mg chromatin	N/A
NELF A	Proteintech	10456-1-AP	AB_2216327	00023389	1:1,000	10ul / 1mg chromatin	N/A
NELF E	Abcam	ab170104	AB_2827280	GR3395420-2	1:1,000	10ul / 1mg chromatin	N/A
SPT6	Cell Signaling Technology	15616S	AB_2798748	Lot 1, 06/2025	1:1,000	10ul / 1mg chromatin	N/A
PAF1	Cell Signaling Technology	12883S	AB_2798052	Lot 1, 05/2025	1:1,000	10ul / 1mg chromatin	N/A
Anti-Rabbit HRP	Millipore Sigma	NA934V	AB_772206	17415553	1:2,000	N/A	N/A
Anti-Mouse HRP	Millipore Sigma	NA931V	AB_772210	17193520	1:2,000	N/A	N/A
Anti-Rabbit Alexa 555	Thermo Fisher	A21428	AB_2535849	2011559	N/A	N/A	1:500

N/A: Not Applicable to this study

Supplemental Table S2. Antibodies

Chemical name	***Supplier***	***Catalog #***
JQ1	Tocris	4499
Birabresib (OTX015)	Selleckchem	S7360
ABBV-744	Selleckchem	S8723
Doxorubicin	Cayman Chemical	15007
Decitabine	Selleckchem	S1200
Doxycycline hyclate	Millipore Sigma	D9891-1G
Puromycin	Millipore Sigma	P8833-25MG
Hygromycin B	Thermo Fisher Scientific	10687010

Supplemental Table S3. Chemicals
